# Pharmacological Inhibition of Ferroptosis as a Therapeutic Target for Neurodegenerative Diseases and Strokes

**DOI:** 10.1002/advs.202300325

**Published:** 2023-06-21

**Authors:** Yumin Wang, Shuang Wu, Qiang Li, Huiyan Sun, Hongquan Wang

**Affiliations:** ^1^ Department of Respiratory and Critical Care Medicine Aerospace Center Hospital Peking University Aerospace School of Clinical Medicine Beijing 100049 P. R. China; ^2^ Department of Neurology Zhongnan Hospital of Wuhan University Wuhan 430000 P. R. China; ^3^ Department of Neurology The Affiliated Hospital of Chifeng University Chifeng 024005 P. R. China; ^4^ Chifeng University Health Science Center Chifeng 024000 P. R. China; ^5^ Tianjin Medical University Cancer Institute and Hospital National Clinical Research Center for Cancer Tianjin's Clinical Research Center for Cancer Key Laboratory of Cancer Prevention and Therapy Tianjin 300060 P. R. China

**Keywords:** Alzheimer's disease, amyotrophic lateral sclerosis, ferroptosis, intracerebral hemorrhage, ischemic stroke, Parkinson's disease, subarachnoid hemorrhage

## Abstract

Emerging evidence suggests that ferroptosis, a unique regulated cell death modality that is morphologically and mechanistically different from other forms of cell death, plays a vital role in the pathophysiological process of neurodegenerative diseases, and strokes. Accumulating evidence supports ferroptosis as a critical factor of neurodegenerative diseases and strokes, and pharmacological inhibition of ferroptosis as a therapeutic target for these diseases. In this review article, the core mechanisms of ferroptosis are overviewed and the roles of ferroptosis in neurodegenerative diseases and strokes are described. Finally, the emerging findings in treating neurodegenerative diseases and strokes through pharmacological inhibition of ferroptosis are described. This review demonstrates that pharmacological inhibition of ferroptosis by bioactive small‐molecule compounds (ferroptosis inhibitors) could be effective for treatments of these diseases, and highlights a potential promising therapeutic avenue that could be used to prevent neurodegenerative diseases and strokes. This review article will shed light on developing novel therapeutic regimens by pharmacological inhibition of ferroptosis to slow down the progression of these diseases in the future.

## Graphical abstract

The roles of ferroptosis contributing to neurodegenerative diseases and strokes are described, and the emerging data in treating these diseases through pharmacological inhibition of ferroptosis, highlighting a potential promising therapeutic avenue that could be used to prevent neurodegenerative diseases and strokes are summarized. This review will shed light on developing novel therapeutic regimens through inhibiting ferroptosis to slow down the progression of these diseases in the future.

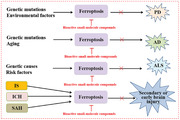

## 1 Introduction

Ferroptosis, a nonapoptotic mode of regulated cell death (RCD), was first described by Dixon in 2012. It is characterized by the iron‐dependent oxidative modification of phospholipid membranes.^[^ ^1^ ^]^ Ferroptosis is triggered by the toxic build‐up of lipid peroxides on cellular membranes through inhibition of the antioxidant defense system and accumulation of iron‐dependent reactive oxygen species (ROS), which react with polyunsaturated fatty acids (PUFAs) and destroy the integrity of cell membranes. The essence of ferroptosis is a new form of RCD driven by highly iron‐dependent lipid peroxidation (LPO) on cellular membranes.^[^ ^2^ ^]^ Ferroptosis, characterized by iron‐dependent LPO and antioxidant defense system dysfunction, is biochemically, morphologically, and genetically different from other forms of RCD which encompass apoptosis, necroptosis, and autophagy. The morphological changes of mitochondria in ferroptotic cells include outer membrane disruption, cristae reduction, shrinkage, and elevation of membrane density.^[^ ^3^ ^]^

Over the past decade, a number of evidence has revealed that ferroptosis play important roles in various diseases, including neurological disorders,^[^ ^4^, ^5^, ^6^, ^7^, ^8^ ^]^ cancers,^[^ ^2^, ^9^ ^]^ metabolic diseases^[^ ^10^, ^11^ ^]^ and cardiovascular diseases.^[^ ^12^ ^]^ Most recently, ferroptosis has garnered enormous interest in research communities of neurological disorders, and has been shown to implicate in pathogenesis of various neurodegenerative diseases (NDs), including Parkinson's disease (PD) and Alzheimer's disease (AD), amyotrophic lateral sclerosis (ALS), and strokes, including acute ischemic stroke (AIS), spontaneous intracerebral hemorrhage (ICH) and subarachnoid hemorrhage (SAH). Furthermore, the pathophysiological relevance of ferroptosis, especially as a therapeutic modality through pharmacological inhibition of ferroptosis to treat and prevent organ damage, has been convincingly established in NDs and strokes, thus opening new opportunities to treat these diseases using pharmacological inhibition of ferroptosis.

This review summarizes recent knowledge about the underlying regulatory mechanism of ferroptosis. Additionally, we discuss the potential pathophysiological roles of ferroptosis in the contribution to NDs and strokes. We review the emerging data from studies in treating NDs and strokes through pharmacological inhibition of ferroptosis. This review article suggests that pharmacological inhibition of ferroptosis by bioactive compounds (ferroptosis inhibitors) as a therapeutic target for NDs and strokes, and highlights a potential promising therapeutic avenue that could be used to negate and counteract NDs and strokes. This article provides a rationale for the development of new neuroprotective bioactive compounds, based on their ability to inhibit ferroptosis to slow down the progression of these diseases.

## 2 Ferroptosis: A Historical Perspective

**Figure 1:**
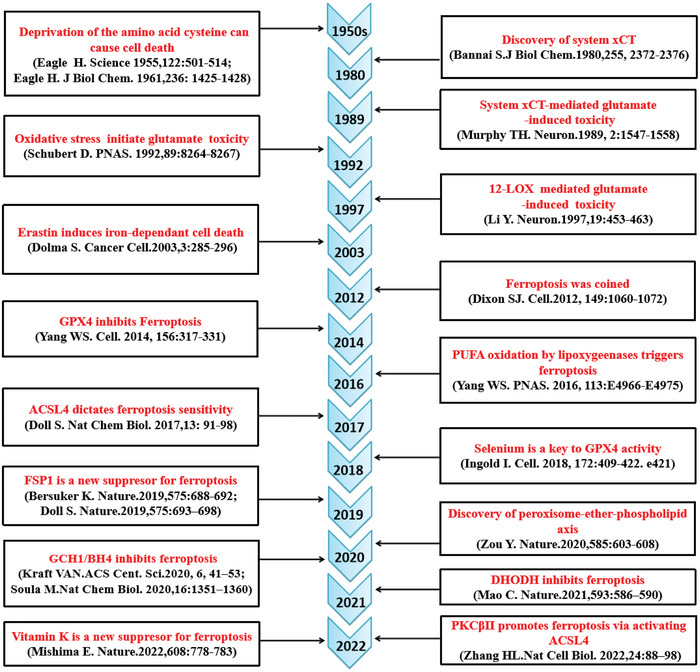
The diagram depicting key milestones in the field of ferroptosis research.

The concept of ferroptosis was introduced by Stockwell lab in 2012 based on three major research areas which provide the foundational understanding of ferroptosis, i.e., the mechanisms of lipid and amino acid metabolism,^[^ ^13^, ^14^, ^15^ ^]^ the control of ROS,^[^ ^16^, ^17^ ^]^ and the regulation of iron^[^ ^18^ ^]^ (**Figure** **1**). The pioneering work by Eagle have shown that deprivation of cysteine (an amino acid) can lead to cell death,^[^ ^13^ ^]^ while endogenous synthesis of cysteine causes cell resistance to this type of cell death.^[^ ^19^, ^20^ ^]^ Ferroptosis was discovered in an attempt to find molecules selectively inducing death in cancer cells carrying an oncogenic form of rat sarcoma virus (RAS) mutant subtypes through high‐throughput screening. During 2001–2003, Stockwell and co‐workers discovered a new compound named as eradicator of RAS‐transformed cells (erastin), which can induce nonapoptotic cell death through a high‐throughput screen in cancer cells carrying an oncogenic form of RAS.^[^ ^21^ ^]^ This pattern of cell death was dependent on the accumulation of cellular iron and oxidative stress and inhibited by iron‐chelating agents.^[^ ^3^ ^]^ Following this discovery, Stockwell and co‐workers performed a larger screen and identified two additional compounds, RAS‐selective‐lethal‐3 small molecule (RSL3) and RSL5, works as synthetic compounds that selectively killed BJeLR cells in a similar iron‐dependent oxidative non‐apoptotic cell death in 2008.^[^ ^22^ ^]^ Almost at the same time, Conrad and co‐workers revealed that genetic ablation of GPX4 resulted in a LPO‐induced non‐apoptotic cell death that could be inhibited by alpha‐tocopherol^[^ ^23^ ^]^ and that enforcing overexpressed the light chain of cystine/glutamate antiporter (system Xc^−^) protected cells from this type of cell death.^[^ ^24^ ^]^ In 2012, Stockwell and co‐workers found that erastin caused an iron‐dependent, non‐apoptotic mode of cell death through inhibiting cystine uptake by system Xc^−^ accompanying with the accumulation of lipid ROS in human fibrosarcoma HT‐1080 cells, involving an unique constellation of biochemical, morphological, and genetic features.^[^ ^1^ ^]^ According to those pioneering works, Stockwell and co‐workers proposed the concept of ferroptosis that was inhibited by a specific inhibitor of ferroptosis, such as ferrostatin‐1(Fer‐1).^[^ ^1^ ^]^

## 3 Molecular Mechanisms of Ferroptosis

**Figure 2:**
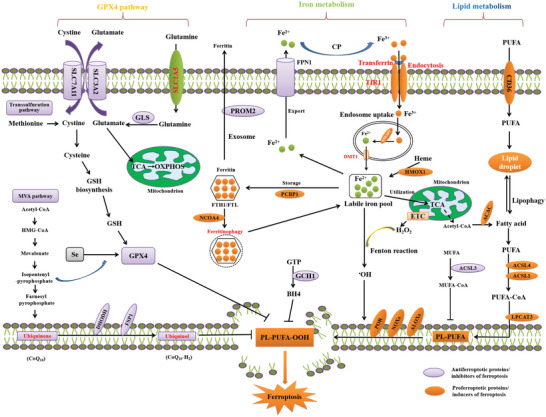
Core mechanisms of ferroptosis. Ferroptosis is mainly caused by iron‐dependent lipid peroxidation. The initiation of ferroptosis requires two key signals, namely the accumulation of free iron and the inhibition of antioxidant SLC7A11/GPX4 system. The generation of polyunsaturated phospholipid (by ACSL4 and LPCAT3) and subsequent activation of ALOX promotes lipid peroxidation. This process requires HO^•^ from an iron‐mediated Fenton reaction or the activation of POR, NOX, or mitochondria electron transport chain pathways. The lipid peroxidation or its secondary products (e.g., 4‐HNE and MDA) induce pore formation in plasma or organelle membrane, which eventually triggers cell death at the final step of ferroptosis. Alternatively, CoQ10 or tetrahydrobiopterin (BH4) inhibits ferroptosis independent of GSH. All aspects of iron metabolism, including iron absorption, storage, export, and utilization, have an important regulatory effect on ferroptosis.

**Figure 3:**
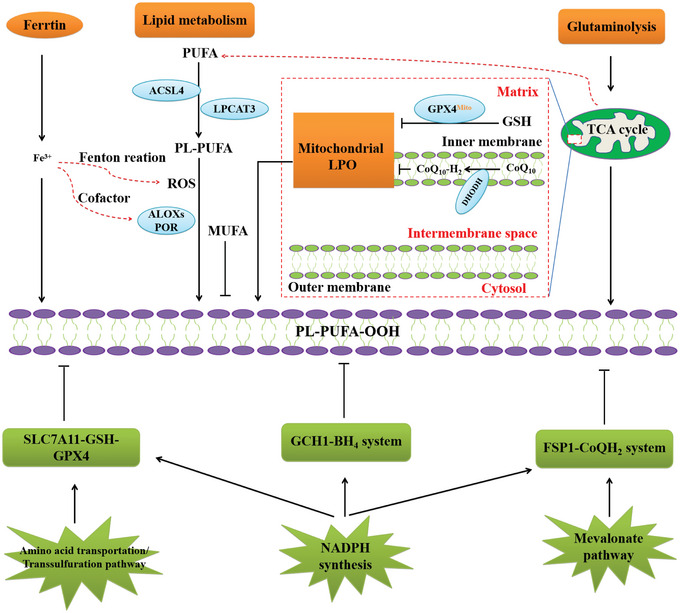
Metabolic processes impinging on the susceptibility of cell toward ferroptosis. Insufficiently controlled intracellular iron storage and PUFA‐enriched phospholipids are prerequisite for the execution of cell death by ferroptosis. Accordingly, MUFAs that compete with PUFAs for incorporation into phospholipids exert antiferroptotic effects. Although mitochondria are dispensable for the execution of ferroptosis, under certain conditions TCA cycle fueled by glutaminolysis may play a major role in ferroptosis induction. Ferroptosis is governed by at least four defense systems with different subcellular localizations to detoxify lipid peroxides and thus protect cells against ferroptosis, i.e., cytosolic GPX4 (GPX4^cyto^) cooperates with FSP1 on the plasma membrane (and other non‐mitochondrial membranes) and mitochondrial GPX4 (GPX4^mito^) cooperates with DHODH in the mitochondria to neutralize lipid peroxides. The SLC7A11/GSH/GPX4 axis, the GCH1/BH4 axis, and the FSP1/CoQ10 axis are fueled by NADPH. Furthermore, the SLC7A11/GSH/GPX4 axis depends on the cystine glutamate antiporter system Xc^−^ and the transsulfuration pathway providing cysteine, while the FSP1/CoQ10 axis relies on the mevalonate pathway that generates CoQ10.

Ferroptosis reflects a delicate antagonistic balance between inducers of ferroptosis and ferroptosis defense systems. When ferroptosis‐promoting factors that include iron‐dependent ROS and LPO significantly override the antioxidant defense systems, a lethal accumulation of lipid peroxides on cellular membranes results in membrane rupture, leading to ferroptotic cell death^[^ ^2^ ^]^ (**Figure** **2**). Ferroptosis defense systems include the SLC7A11‐reduced glutathione (GSH)‐glutathione peroxidase 4 (GPX4) system, the dihydroorotate dehydrogenase‐dihydroubiquione (DHODH‐CoQH2) system, the ferroptosis suppressor protein 1‐ubiquinol (FSP1‐CoQH2) system, and the GTP cyclohydrolase 1‐tetrahydrobiopterin (GCH1‐BH4) system.^[^ ^2^ ^]^ Ferroptosis is mainly driven by iron accumulation and LPO, and subsequent plasma membrane rupture.^[^ ^25^ ^]^ The induction of ferroptosis needs two key signals, namely the accumulation of free iron and the inhibition of antioxidant SLC7A11‐GSH‐GPX4 system.^[^ ^26^ ^]^ There are many connections between lipid reactive oxygen species and each metabolic process (**Figure** **3**).

### 3.1 Iron Homeostasis

Iron, an essential redox‐active metal for normal physiological functions, is important for neuronal activity and plays an important role in energy metabolism, neurotransmitter biosynthesis, and myelination.^[^ ^27^ ^]^ In the human body, the cellular iron metabolism is regulated by transport, absorption and recycling of iron. At the transcriptional level, iron was regulated by iron response elements (IRE) and iron regulatory proteins (IRP),^[^ ^28^ ^]^ avoiding the accumulation of iron leading to iron overload, which is highly harmful to tissues.^[^ ^8^, ^29^ ^]^ Iron homeostasis is highly regulated and is crucial to maintain its normal function in the brain.^[^ ^28^ ^]^ The dysregulation of iron within the brain causes oxidative stress‐dependent injury to neurons, leading to neurological diseases. Iron‐dependent ROS and LPO are of great significance in the regulation of ferroptosis. Iron is involved in free radical formation and propagation of LPO.

The transferrin receptor 1 (TfR1), which is expressed on the luminal side of the brain microvascular endothelial cells (BMECs), is responsible for regulating the content of iron transported into the brain through the blood‐brain barrier (BBB) to maintain iron homeostasis for proper brain function.^[^ ^30^ ^]^ The iron (Fe^3+^) binds to circulating transferrin (Tf) to form a complex holo transferrin (holo‐Tf),^[^ ^31^ ^]^ which binds to TfR1 and then enters into the BMECs through endocytosis mediated by clathrin.^[^ ^32^ ^]^ The Fe^3+^‐Tf‐TfR complex is then transported into cells to the endosome where Fe^3+^ detaches from Tf, and is reduced to Fe^2+^ by metalloreductases six‐transmembrane epithelial antigen of prostrate 3 (STEAP3)^[^ ^33^ ^]^ or duodenal cytochrome b (DCYTB).^[^ ^34^ ^]^ Fe^2+^ then enters the cytosol by divalent metal transporter 1 (DMT‐1).^[^ ^35^ ^]^ The unbound redox‐active iron (Fe^2+^) in the cytosol constitutes a labile iron pool (LIP), which acts as an intermediate between stored, utilized, or imported iron, feeds iron‐catalyzed ROS production.^[^ ^36^ ^]^ The utilized unbound Fe^2+^ can be transported to the mitochondrion for the biosynthesis of heme, formation of iron‐sulfur clusters, and as co‐factors for mitochondrial enzymes. Also, the unbound Fe^2+^ can be stored in a non‐toxic form as Fe^3+^ by ferritinin, which consists of two isoforms, i.e. heavy (FTH) and light (FTL). FTH has ferroxidase activity, involving in rapid iron uptake and reutilization. FTL plays a role in long‐term storage of iron through nucleation.^[^ ^27^, ^28^, ^37^, ^38^ ^]^ Ferritin can be degraded through ferritinophagy, an autophagy‐like process by which releases labile iron and facilitates LPO driving ferroptosis.^[^ ^39^ ^]^ The unbound excess Fe^2+^ can be exported from the cells via ferroportin (FPN) with the help of ceruloplasmin or hephaestin to oxidize Fe^2+^ to form Fe^3+^.^[^ ^40^, ^41^ ^]^

Cellular iron homeostasis is maintained by iron regulatory proteins (IRP1 and IRP2),^[^ ^42^, ^43^ ^]^ which bind to the iron response element (IRE) of iron metabolizing genes and regulate their expression, leading to changes in cellular iron metabolism.^[^ ^44^, ^45^ ^]^ When intracellular iron is low, Fe–S is released from the active site of the IRP, permitting IRP binds to IRE of DMT1 and TfR gene transcripts to activate their translation, while IRPs bind to FPN gene transcripts to inhibit its translation, resulting in promoting absorption of free iron to increase intracellular dose and reducing cellular iron excretion.^[^ ^8^, ^29^, ^46^ ^]^

The accumulation of free iron is involved in the accumulation of lethal lipid peroxides and the initiation of ferroptosis.^[^ ^26^ ^]^ Iron catalyzes the Fenton reaction or acts as an essential cofactor for cytochrome P450 oxidoreductase (POR) or arachidonate lipoxygenases (ALOXs), which promote LPO.^[^ ^2^ ^]^ The Fenton reaction induced the generation of free radicals that attack membrane‐bound PUFAs in a nonenzymatic pathway, while nonheme iron‐containing lipoxygenases (LOXs) initiate the dioxygenation of membrane phospholipids containing PUFAs, i.e. linoleic acid (LA, 18:2 n‐6) to arachidonic acid (AA, 20:4 n‐6) in the enzymatic LPO pathway.^[^ ^47^ ^]^ Due to its ability of oxidation‐reduction, the iron (Fe^2+^) converts hydrogen peroxide (H_2_O_2_) to toxic hydroxyl radicals (HO^•^), an important ROS acting on LPO, resulting in oxidative stress and cell death via the Fenton and Haber‐Weiss reaction, known as: Fe^2+^ + H_2_O_2_ → Fe^3+^ + OH^•^ + OH^−^, first described in 1894.^[^ ^48^ ^]^ The free radicals generated near membranes react with PUFAs, i.e., initiate peroxidation of PUFAs, producing lipid hydroperoxides which are highly susceptible to breakdown, forming a variety of lipid‐derived *α*,*β*‐unsaturated 4‐hydroxyaldehydes, of which the most prominent is 4‐hydroxynonenal (HNE).^[^ ^49^, ^50^ ^]^ Meanwhile, iron can promote the activity of iron‐dependent peroxidases including lipoxygenases (LOXs) and prolyl hydroxylase domain (PHD), thereby increasing the sensitivity of cells to ferroptosis.^[^ ^51^ ^]^

### 3.2 Lipid Peroxidation

During ferroptosis, the substrates for LPO are PL‐PUFAs due to their intrinsic susceptibility to peroxidation chemistry.^[^ ^39^ ^]^ The PL‐PUFAs are generated by different enzymes such as acyl‐coenzyme A [CoA] synthetase long‐chain family member 4 (ACSL4) and lysophosphatidylcholine acyltransferase (LPCATs). In nonenzymatic LPO, PUFAs are ligated with CoA by ACSL4 to produce acyl‐CoA, which can be re‐esterified in phospholipids through LPCATs to produce PL. PUFAs can be scavenged from the environment and dietary sources and can be synthesized from the basic building block acetyl CoA, through the action of acetyl CoA carboxylase (ACC).^[^ ^39^ ^]^ Once polyunsaturated acyl tails (PL‐PUFAs) are incorporated into membrane environments, ALOXs and cytochrome P450 oxidoreductase (POR), and labile iron use molecular oxygen (O_2_) to do a peroxidation reaction, leading to the generation of peroxidated PL‐PUFAs (PL‐PUFA‐OOH).^[^ ^39^, ^52^ ^]^ This process requires hydrogen peroxide (H_2_O_2_) derived from an iron‐dependent Fenton reaction, or POR and NADPH oxidase (NOX) activation, or mitochondria electron transport chain pathways. The last step of ferroptosis, LPO or its secondary products such as 4‐HNE and MDA cause pore formation in plasma or organelle membranes, eventually triggering cell death. In recent years, ferroptosis has gained substantial attention and sparked great interest in the research community of neurological diseases, and targeting ferroptosis through pharmacological inhibition might provide new therapeutic opportunities to treat neurological diseases.^[^ ^53^, ^54^ ^]^

### 3.3 Ferroptosis Defense Systems

#### 3.3.1 SLC7A11‐GSH‐GPX4 Axis

The cellular antioxidant systems that directly neutralize lipid peroxides is one of the ferroptosis defense systems. There exist four anti‐ferroptosis defense systems with distinctive subcellular localizations. The SLC7A11‐GSH‐GPX4, which is related to amino acid metabolism, is well‐defined and believed to constitute the major cellular system to protect against ferroptosis.^[^ ^2^, ^8^ ^]^ Solute carrier family 7 member 11 (SLC7A11; also known as xCT) and solute carrier family 3 member 2 (SLC3A2) consists of the system Xc^−^.^[^ ^55^, ^56^ ^]^ xCT, the transporter subunit, is a core component of system Xc^−^. xCT import extracellular cystine and export intracellular glutamate to mediate the antiporter activity of system Xc^−^.^[^ ^55^, ^57^ ^]^ The extracellular cystine taken up by SLC7A11 is rapidly reduced to cysteine through a nicotinamide adenine dinucleotide phosphate (NADPH)‐consuming reduction reaction in the cytosol. Then cysteine serves as the rate‐limiting precursor for the biosynthesis of GSH, which is a principle cofactor for GPX4‐mediated LPO detoxification.^[^ ^56^ ^]^ Blocking transporter activity of SLC7A11 or depleting cystine in culture media can induce ferroptosis in various cancer cells.^[^ ^56^ ^]^ GPX4 belongs to the GPX protein family with activity of lipid repair enzyme,^[^ ^58^, ^59^ ^]^ and was identified as a key inhibitor in ferroptosis.^[^ ^1^, ^60^, ^61^ ^]^ GPX4 can convert PL hydroperoxides (L‐OOH) to non‐toxic lipids PL alcohols and simultaneously oxidize two GSH to oxidized glutathione (GSSG).^[^ ^16^, ^62^ ^]^ Previous studies have established the critical role of GPX4 in ferroptosis suppression through genetic or pharmacological manipulation.^[^ ^63^, ^64^ ^]^ GPX4 is regulated by epigenetic, transcriptional, and post‐translational modifications (PTMs) such as phosphorylation, ubiquitination, succination, and glycosylation.^[^ ^65^ ^]^

#### 3.3.2 The FSP1‐CoQH_2_ System

Coenzyme Q_10_ (CoQ_10_) is found in diverse membranes throughout cells, including mitochondria, and serves as a second endogenous mechanism for protecting membranes against lipid peroxidation that drives ferroptosis. The ferroptosis suppressor protein 1 (FSP1; also known as AIFM2^[^ ^66^ ^]^) was firstly identified to operate independently of GPX4 to defend against ferroptosis.^[^ ^67^, ^68^ ^]^ FSP1 localizes on the plasma membrane (as well as on other subcellular compartments) and its plasma membrane localization appears to be both necessary and sufficient for the function of FSP1 in suppressing ferroptosis.^[^ ^67^, ^68^ ^]^ FSP1 functions as an NAD(P)H‐dependent oxidoreductase to reduce ubiquinone (also known as coenzyme Q_10_) to regenerate the reduced form of CoQ_10_ ubiquinol (CoQ_10_‐H_2_), which can trap lipid peroxyl radicals, thereby suppressing lipid peroxidation and ferroptosis.^[^ ^50^, ^51^ ^]^ In some cases, FSP1 inhibits ferroptosis by activating ESCRT‐III‐dependent membrane repair instead of its oxidoreductase function.^[^ ^69^ ^]^ The ESCRT III complex repairs damage to the plasma membrane and slows cell death by ferroptosis.^[^ ^70^ ^]^

#### 3.3.3 The GCH1‐BH_4_ System

Recent studies have shown that GTP cyclohydrolase 1 (GCH1)‐tetrahydrobiopterin (BH_4_) system functions as another critical GPX4‐independent suppressor of ferroptosis through suppressing lipid peroxidation.^[^ ^71^, ^72^ ^]^ BH_4_ is a cofactor of aromatic amino acid hydroxylases and other enzymes, and GCH1 mediates the rate‐limiting reaction in the BH_4_ biosynthesis pathway. GCH1, which generates the endogenous metabolite BH_4_, was discovered in a CRISPR activation screen as a suppressor of ferroptosis,^[^ ^71^ ^]^ as well as an enhancer of ferroptosis in a CRISPR loss‐of‐function screen.^[^ ^72^ ^]^ GCH1 suppresses ferroptosis through the generation of BH_4_ as a radical‐trapping antioxidant as well as via GCH1‐mediated production of CoQH_2_ and PL‐PUFAs.

#### 3.3.4 The DHODH‐CoQH_2_ System

The DHODH‐CoQH_2_ system is a newly identified GPX4‐independent mitochondria‐localized ferroptosis defense that can compensate for GPX4 loss to detoxify mitochondrial lipid peroxidation.^[^ ^73^ ^]^ DHODH, an enzyme involved in pyrimidine synthesis that can reduce CoQ10 to CoQH_2_ in the inner mitochondrial membrane, functions by reducing mitochondrial CoQ10, analogous to the function of FSP1 in extra mitochondrial membranes.^[^ ^73^ ^]^ Following the inactivation of GPX4, the flux through DHODH is significantly increased, leading to promoting CoQH_2_ generation, which neutralizes lipid peroxidation and inhibits ferroptosis in mitochondria.^[^ ^73^ ^]^ Inactivation of both GPX4 and DHODH in mitochondria triggers robust ferroptosis through unleashing potent mitochondrial lipid peroxidation. Cells with a low expression of GCH1 or DHODH confer more sensitivity to ferroptosis, and those with high expression confer resistance to ferroptosis.

## 4 Ferroptosis in Neurological Diseases

### 4.1 Ferroptosis in Neurodegenerative Diseases

#### 4.1.1 Parkinson's Disease

**Figure 4:**
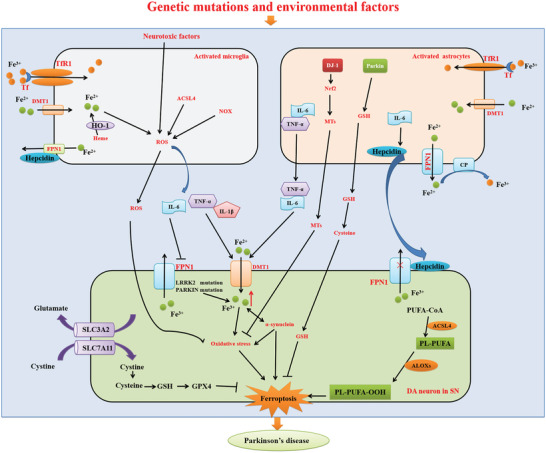
Potential molecular mechanisms of ferroptosis in PD development. The activated microglia and astrocytes release the inflammatory cytokines (IL‐1*β*, IL‐6, and TNF‐*α*), which promote iron accumulation in neurons by downregulating FPN1 and upregulating DMT1. IL‐6 promotes the release of hepcidin from astrocytes and thus prevents iron release from neurons mediated by FPN1. Activated microglia release ROS to promote neuronal oxidative stress. Upregulation of Nrf2 and the release of MTs in astrocytes protect neuron against oxidative stress. Two types of glia regulate glutamate‐mediated toxicity and influence ferroptosis. Astrocytes effectively control the level of glutamate in the synaptic cleft by regulating GSH synthesis and inhibiting neuronal xCT. Activated microglia release TNF‐*α*, promoting neuronal glutamine release. The gene mutations of LRRK2 and PARKIN contribute to excess iron accumulation in PD. The accumulation of iron potentiates *α*‐synuclein‐mediated neurotoxicity. The *α*‐synuclein induces oxidative stress and lipid peroxidation.

Parkinson's disease (PD) is a neurodegenerative disease characterized by loss of dopaminergic neurons in the substantia nigra and the classical motor features of parkinsonism are associated with Lewy bodies.^[^ ^74^ ^]^ The pathogenesis of PD remains largely unknown, but seems to develop as a result of complex interaction among multiple predisposing genes and environmental factors.^[^ ^75^ ^]^ The oxidative stress hypothesis of PD holds that oxidative stress leads to the neurodegeneration of dopaminergic neurons and results in the pathogenesis of PD, which was proposed in 1992, based on the observation that increased oxidative stress by PD‐related neurotoxins, increased LPO and decreased GSH, increased iron and reduced ferritin and decreased activities of glutathione peroxidase and catalase of in the SN of patients with PD.^[^ ^76^ ^]^ The PD pathological hallmarks include iron dyshomeostasis, LPO, GSH depletion, and oxidative stress are all related to ferroptosis. After coined the term ferroptosis, which is characterized by iron dysregulation, LPO, and GSH depletion, scientists begin to revisit the contributions of aforementioned signatures of PD to the disease development, and a ferroptosis hypothesis of PD is emerging, which claims that ferroptosis is involved in the pathogenesis of PD^[^ ^47^, ^77^, ^78^, ^79^, ^80^ ^]^ (**Figure** **4**). The direct relationship between ferroptosis and PD was confirmed in vitro in organotypic slice cultures and in vivo in the MPTP mouse model.^[^ ^81^, ^82^ ^]^

##### Role of Iron and Ferroptosis in PD

Iron dys‐homeostasis and accumulation contribute to progression of neuropathology through ROS production and are a common feature of PD.^[^ ^47^ ^]^ Ample evidence has shown that increased iron in glia and dopaminergic neurons in the substantia nigra pars compacta (SNpc) in PD^[^ ^83^, ^84^, ^85^, ^86^, ^87^, ^88^ ^]^ is associated with disease severity.^[^ ^89^, ^90^ ^]^ The microglial iron and ferritin are elevated in the SN in the animal models of PD induced by 1‐methyl‐4‐phenyl‐1,2,3,6‐tetrahydropyridine (MPTP) and 6‐hydroxydopamine (6‐OHDA).^[^ ^91^ ^]^ The iron accumulation in PD probably results from the dysregulation of IRP triggered iron control homeostatic mechanisms, resulting in increased intracellular iron import or decreased export, which constitute vulnerability factors for PD development.^[^ ^81^, ^92^, ^93^, ^94^ ^]^ PD patients with mutations in iron‐bound proteins are more prone to develop PD.^[^ ^92^, ^93^, ^94^, ^95^ ^]^ Elevated DMT1 levels in the SNpc of PD patients and PD mouse models are likely related to an increased cellular iron import.^[^ ^96^, ^97^ ^]^ It has been shown that the levels of iron storage protein ferritin decreased in the SN from post‐mortem brains of patients with PD and PD models,^[^ ^98^ ^]^ and ferroportin (iron efflux protein) is decreased in MPTP or 6‐OHDA‐induced PD models.^[^ ^99^, ^100^, ^101^ ^]^ Ferritin, the main iron storage protein important in maintaining iron homeostasis, confers protection against iron‐mediated ferroptosis through chelating iron.^[^ ^102^ ^]^ Astrocytes increased ferritin release to respond to iron overload, which might inhibit iron‐mediated oxidative damage and ferroptosis of dopamine (DA) neurons in PD.^[^ ^102^ ^]^ SEC24B‐regulated ferroptosis in microglia contributes to neurodegeneration in PD.^[^ ^103^ ^]^ The microglia grown in a human induced pluripotent stem cell (iPSC)‐derived triculture system that contains microglia, neurons, and astrocytes are highly responsive to iron and susceptible to ferroptosis. Removal of microglia from the tri‐culture system substantially delayed iron‐induced neurotoxicity, suggesting a critical role for microglia iron overload and ferroptosis in neurodegeneration in PD patients.^[^ ^103^ ^]^ Taken together, there exists a dysregulation of iron influx and efflux mechanisms in PD, which contribute to the accumulation of intracellular iron that causes susceptibility of iron‐mediated free radical formation and ferroptosis.

##### LPO and Ferroptosis in PD

The oxidative stress and iron dependent increased LPO leading to nigral cell death was first reported by Dexter in 1986.^[^ ^104^ ^]^ Postmortem analyses of brain found that PUFAs, but not MUFAs, decreased in the SN of PD patients.^[^ ^104^ ^]^ The levels of MDA were elevated in SN^[^ ^105^ ^]^ and levels of lipid hydroperoxide (LOOH) in plasma is increased.^[^ ^106^ ^]^ A previous study showed that 4‐HNE was associated with Lewy bodies in the SN of PD patients.^[^ ^107^ ^]^ The elevated 4‐HNE in the cerebrospinal fluid (CSF) of PD patients is positively correlated with an increase of iron in the SN.^[^ ^107^, ^108^ ^]^ Recent studies have demonstrated that LPO is increased in PD in vitro and in vivo models induced by rotenone,^[^ ^109^ ^]^ 6‐OHDA,^[^ ^110^, ^111^, ^112^, ^113^ ^]^ MPP^+^,^[^ ^114^ ^]^ and MPTP.^[^ ^115^ ^]^ Another study showed a direct association between endogenous *α*‐synuclein (*α*‐syn) levels and the sensitivity of dopaminergic neurons to lipid peroxidation and ferroptosis via modulation of plasma membrane ether‐linked phospholipids.^[^ ^116^ ^]^ This study uncovered that *α*‐syn functions as a positive modulator of ferroptosis, supporting ferroptosis as a key mechanism involved in the pathology of PD and providing potential ferroptosis‐based therapeutic opportunities in PD.^[^ ^116^ ^]^ This observation was supported by other studies, which demonstrated that *α*‐syn overexpression sensitizes neuronal cells to ferroptosis induction through suppressing Nrf2 protein.^[^ ^117^ ^]^ Arachidonic acid (AA) cotreatment with iron, increases AA‐containing phospholipid and synergistically increased the susceptibility of dopaminergic neurons to ferroptosis through promoting high lipid peroxidation, which can be rescued by Ferrostatin‐1. Inhibiting ACSL4 and lipoxygenases 15/15B attenuate dopaminergic neuronal cell death.^[^ ^118^ ^]^ The mutations of calcium‐independent phospholipase A_2_ *β* (iPLA_2_ *β*, PLA2G6, or PNPLA9 gene) are likely involved in PD pathogenesis. iPLA_2_ *β* plays a critical part in ferroptosis by regulating the metabolism of PUFAs. iPLA_2_ *β* can act as an antiferroptotic guardian by eliminating the proferroptotic signal. Deficiency in iPLA_2_ *β*, caused by genetic factors or chemical or pharmacological poisoning, may be associated with increased sensitivity to ferroptotic cell death in PD.^[^ ^119^ ^]^

##### The Role of Inhibition of GSH/GPX4 Axis in PD

Decreased GSH metabolism and a decreased GSH/GSSG ratio are associated with oxidative stress in PD.^[^ ^120^, ^121^ ^]^ GPX4 is decreased in the SN of PD patients and increased in the surviving dopaminergic neurons, indicating that upregulation of GPX4 may act as a compensatory mechanism to protect surviving neurons against ferroptosis‐induced injury during disease progression.^[^ ^122^ ^]^ Meanwhile, the expression of SLC7A11 is downregulated in the SN of PD patients, which may contribute to a reduced GSH levels in the SN of PD.^[^ ^123^ ^]^ Paraquat (PQ), a neurotoxicant which is linked to increased PD risk and PD‐like neuropathology, can promote neurotoxicity of dopaminergic neurons through downregulating SLC7A11/GPX4, upregulating Cox2 expression, and causing iron accumulation through ferritinophagy pathway induced by NCOA4.^[^ ^124^ ^]^ Recent studies have revealed that PD‐related neurotoxins downregulate the expression of SLC7A11 by rotenone^[^ ^109^ ^]^ and MPP^+^,^[^ ^114^ ^]^ and downregulate the expression of GPX4 by rotenone,^[^ ^109^ ^]^ 6‐OHDA,^[^ ^110^, ^112^ ^]^ and MPP^+[^ ^125^ ^]^ in PD in vitro and in vivo models.

##### The Role of FSP1‐CoQH_2_ System in PD

An iron‐free form of ferritin, apoferritin improves motor deficits in MPTP treated mice by inhibiting iron aggregation through down‐regulating iron importer divalent metal transporter 1 (DMT1) and ferroptosis, which was resulting from effectively down‐regulating ACSL4 and the up‐regulating FSP1.^[^ ^126^ ^]^

##### The Role of GCH1‐BH_4_ System in PD

GCH1 catalyzes the rate‐limiting step in tetrahydrobiopterin (BH4) synthesis, an essential cofactor in the synthesis of monoaminergic neurotransmitters, including dopamine (DA). It was found that the level of BH4 and activity of GCH1 were markedly decreased in the substantia nigra and striatum of PD patients.^[^ ^127^, ^128^ ^]^ Loss‐of‐function mutations in GCH1 lead to striatal DA depletion and nigrostriatal cell loss.^[^ ^129^ ^]^ Mounting genetic association studies have also identified that GCH1 variants increased the risk for PD.^[^ ^129^, ^130^, ^131^, ^132^ ^]^ The putative damaging variants of GCH1 contributed to the collective risk for early‐onset Parkinson's disease (EOPD) in ethnic Chinese.^[^ ^133^ ^]^ An early study reported a lower BH4 concentration in the CSF of PD patients.^[^ ^134^, ^135^ ^]^ These studies suggest that there exist an impairment of GCH1‐BH4 system in PD. Most recent study revealed a mechanism that GCH1 deficiency may contribute to PD. *GCH1* ^−/−^ zebrafish develop a marked deficiency of monoaminergic neurotransmitter. Tyrosine hydroxylase (Th) protein levels were markedly reduced. L‐DOPA treatment improved survival without ameliorating the motor phenotype in *GCH1* ^−/−^ larvae. Microglial activation was found in *GCH1* ^−/−^ larvae. These results suggest that GCH1 deficiency may unmask early, subclinical parkinsonism and only indirectly contribute to neuronal cell death via immune‐mediated mechanisms.^[^ ^136^ ^]^

##### The Role of PD‐Related Regulator in PD

Specific regulators play a role in modulating ferroptosis and PD. 6‐OHDA upregulates super‐enhancer‐driven sorting nexin 5 in PD rats. Super‐enhancer‐driven sorting nexin 5 promotes dopaminergic neuronal ferroptosis in 6‐OHDA‐lesioned SNc of PD rats.^[^ ^137^ ^]^ Silencing SNX5 significantly inhibit ferroptosis in 6‐OHDA‐induced PC12 cells, suggesting the correlation between the SNX5, ferroptosis, and PD. Overall, SNX5 functions as an inducer of ferroptosis in PD.^[^ ^137^ ^]^ This observation was corroborated by study from the same group, which reported SNX5 promotes ferroptosis in the PD model.^[^ ^138^ ^]^ Mutations and increased leucine‐rich repeat kinase 2 (LRRK2) kinase activity are associated with both familial and idiopathic PD pathology. The wild‐type cells were more resistant to ferroptosis than the LRRK2 knockout RAW 264.7 murine macrophages. Inhibition of the LRRK2 kinase increased the sensitivity of cell to erastin. These results suggest that LRRK2 protects against erastin‐induced ferroptosis in RAW macrophages.^[^ ^139^ ^]^

#### 4.1.2 Alzheimer's Disease

**Figure 5:**
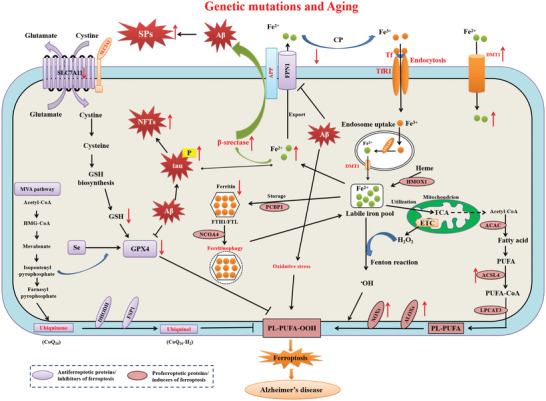
Schematic representation of molecular mechanisms of ferroptosis in Alzheimer's disease. The dyshomeostasis of iron in AD. Aging, inflammation, and oxidative stress could dysregulate the iron transport proteins and cause iron retention. Increased irons could be exported by FPN1/Cp or FPN1/Heph with the help of APP, which is transported to stabilize FPN1 by soluble tau protein. The overload Fe^2+^ could upregulate the expressions of ferritin, FPN1, and APP by IRP‐IRE interactions, while suppress the function of furin, thereby enhancing *β*‐secretase activity by reducing furin protein expression and thus accelerating A*β* production and deposition. Aberrant phosphorylation of the tau protein can lead to increased APP and A*β*40 aggregation. A*β* directly down‐regulate FPN and decrease levels of GPX4 and elevate levels of ferritin. Excessive iron in neurons can lead to tau hyperphosphorylation and NFT formation. When xCT or GSH decreases in neurons, the decreased GPX4 cannot exert the function of anti‐lipid peroxidation. After Fenton reaction or ALOX‐catalyzed process, PUFA‐OOH can accumulate to a lethal level to trigger ferroptosis, which could be responsible for the tau hyperphosphorylation, A*β* formation, and neuronal loss.

Alzheimer's disease (AD) is the most common neurodegenerative disease characterized by cerebral atrophy, accumulation of amyloid beta‐peptide (A*β*) in senile plaques (SPs), and intraneuronal accumulation of hyperphosphorylated microtubule ‐associated protein tau in neurofibrillary tangles (NFTs), resulting in a progressive decline in cognitive function.^[^ ^140^ ^]^ The iron dyshomeostasis, LPO, GSH depletion, and oxidative stress are implicated in the pathogenesis of AD.^[^ ^141^, ^142^, ^143^ ^]^ After the introduction of the term ferroptosis, which is characterized by iron dysregulation, LPO, and GSH depletion, the science community begins to revisit the contributions of aforementioned signatures of AD to the disease development, and proposed"Ferroptosis hypothesis in Alzheimer's disease," which claims that ferroptosis is involved in the pathogenesis of AD.^[^ ^144^, ^145^, ^146^, ^147^, ^148^ ^]^ The involvement of ferroptosis in the pathogenesis of AD result from iron metabolism dysregulation, LPO, and the inhibition of GSH‐GPX4 axis (**Figure** **5**).

##### The Role of Iron Metabolism Dysregulation in AD

Elevated iron in the brains of patients with AD was first revealed in 1953.^[^ ^141^ ^]^ This observation was supported by subsequent post‐mortem^[^ ^84^, ^149^, ^150^, ^151^, ^152^ ^]^ and in vivo^[^ ^145^, ^149^, ^152^] studies. Ample evidence has shown that aberrant levels of iron regulatory molecules, such as elevated CSF ferritin,^[^ ^153^ ^]^ hepcidin,^[^ ^154^ ^]^ Tf (iron transport),^[^ ^155^, ^156^ ^]^ ferritin (iron storage), and decreased ferroportin (Fpn, iron export),^[^ ^157^, ^158^ ^]^ are associated with the pathogenesis of AD. Elevated iron occurs even at the early stage as mild cognitive impairment of AD^[^ ^159^, ^160^ ^]^ and result in cognitive decline prior to the disease.^[^ ^161^, ^162^ ^]^ Excess free iron are found within SPs, which upregulates ferritin expression, and impairs spatial memory in AD mouse models.^[^ ^163^, ^164^ ^]^ A*β* is generated from amyloid precursor protein (APP), a single‐pass transmembrane protein that helps protect cells from iron‐mediated oxidative stress by loading Fe^3+^ onto transferrin and stabilizing FPN.^[^ ^152^ ^]^ APP knockout mice exhibited decreased FPN and increased iron accumulation with concurrent oxidative stress in cortical neurons.^[^ ^152^ ^]^ High level of iron upregulates APP translation, which is modulated by an IRE. Iron binds to and promotes toxicity of A*β* and tau.^[^ ^165^, ^166^, ^167^, ^168^, ^169^, ^170^ ^]^ Iron overload injures microglia through increasing ROS production. Meanwhile, some ferroptosis inhibitors, such as vitamin E and desferrioxamine (DFE) have shown clinical benefits for the patients with AD.^[^ ^171^, ^172^ ^]^ Loss of Fpn induces memory impairment by promoting ferroptosis in AD.^[^ ^173^ ^]^ Fpn was downregulated in the brains of APPswe/PS1dE9 mice as an AD mouse model and AD patients. Deletion of Fpn in mice led to AD‐like hippocampal atrophy and memory deficits. Inhibitors of ferroptosis effectively reduced the neuronal death and memory impairments induced by A*β* aggregation in vitro and in vivo. Restoring Fpn ameliorated ferroptosis and memory impairment in APPswe/PS1dE9 mice.^[^ ^173^ ^]^ Decreased FTH1 and SAT1 resulted in a decrease in the accumulation of lipid ROS and eventually result in ferroptosis of astrocytes in AD, suggesting that activated ferroptosis in astrocytes may contribute to the pathophysiological process in the entorhinal cortex in AD.^[^ ^174^ ^]^ Recent study showed that A*β*_1‐40_ caused blood‐brain barrier (BBB) disruption by upregulating CD36 expression in pericytes via inducing pericyte mitophagy‐dependent ferroptosis through the CD36/PINK1 /Parkin pathway, uncovering a molecular mechanism by which pericytes of the BBB disrupted in AD.^[^ ^175^ ^]^ A*β* caused spatial learning and memory impairment, along with intracellular A*β* deposits, which were reversed by treatment with Fer‐1. A*β* decreased the expression of GPX4 and SLC7A11 and increased the level of TfR, suggesting ferroptosis are involved in A*β* neurotoxicity.^[^ ^176^ ^]^ ORMDL3 gene, a susceptibility gene closely related to the occurrence of childhood asthma, promote AD through inducing ferroptosis by PERK/ATF4/HSPA5 signaling pathway.^[^ ^177^ ^]^

##### The Role of LPO in AD

The mammalian brain is inadequately equipped with antioxidant defense systems and is prone to oxidative damage due to its high O_2_ consumption, and neuronal membrane lipids rich in high PUFAs side‐chains, especially DHA (C22:6) residues,^[^ ^178^ ^]^ Iron is found throughout the brain,^[^ ^28^, ^179^, ^180^ ^]^ neuronal mitochondria generate O_2_^·−^, and brain metabolism generates a lot of H_2_O_2_.^[^ ^178^ ^]^ LPO can generate some aldehyde byproducts, including 4‐HNE, malondialdehyde (MDA), and acrolein.^[^ ^181^ ^]^ Previous studies have shown increased LPO and decreased PUFAs in the brain of patients with AD, and increased 4‐HNE in ventricular fluid, among other markers of oxidative stress.^[^ ^143^, ^182^, ^183^, ^184^ ^]^ Mounting evidence indicates elevated levels of 4‐HNE^[^ ^185^, ^186^, ^187^ ^]^ and acrolein^[^ ^186^, ^187^ ^]^ in AD brains. The increased 4‐HNE and acrolein also occur in MCI and early stage of AD, suggesting that LPO is an early event in the pathogenesis of AD.^[^ ^186^, ^187^ ^]^ 4‐HNE exert cytotoxicity to neurons through increasing Ca^2+^ levels, damaging neurofilament proteins, and inactivating glutamate transporters.^[^ ^188^, ^189^ ^]^ Co‐localization of A*β* plaques and LPO products were observed in the brain of AD patients.^[^ ^190^ ^]^ A*β* peptides can induce LPO, which increases APP processing, resulting in a vicious circle between A*β* and LPO in AD pathology.^[^ ^191^ ^]^ Previous studies indicate that some enzymes responsible for LPO, including LOXs,^[^ ^192^, ^193^, ^194^ ^]^ COXs,^[^ ^195^, ^196^ ^]^ and NOXs,^[^ ^197^, ^198^ ^]^ are increased in AD^[^ ^199^, ^200^ ^]^ and play vital roles in the pathogenesis of AD. Iron‐chelating agents such as desferrioxamine can largely inhibit LPO of isolated brain tissues.^[^ ^201^ ^]^ Pharmacologically inhibiting 12/15‐LOX can reverse AD‐like phenotype in a mouse model.^[^ ^199^ ^]^ A recent study has shown that NADPH oxidase 4 (NOX4), a major source of ROS, was significantly elevated in impaired astrocytes of the cerebral cortex from patients with AD and the APP/PS1 double‐transgenic mouse model of AD. Overexpression of NOX4 promotes cytotoxicity by inducing ferroptosis through the activation of lipid peroxidation in human astrocytes.^[^ ^202^ ^]^ In general, dysregulation of LPO and perturbation of lipid metabolism are involved in the pathophysiology of AD.

##### The Role of Inhibition of GSH/GPX4 Axis in AD

GSH is the most abundant nonprotein thiol to exert antioxidant defense role and maintain redox homeostasis in neurons. The depletion of GSH in the brain is a common finding and is linked to loss of neurons, resulting in neurological diseases such as PD, stroke, and AD.^[^ ^203^, ^204^ ^]^ GSH maintains redox homeostasis through binding to Fe^2+^ in the LIP to inhibit the iron‐dependent oxidization^[^ ^205^ ^]^ and functions as the substrate of GPX4‐mediate lipid detoxification.^[^ ^63^, ^64^ ^]^ Decreased levels of GSH in the brain were found in both animal models^[^ ^206^, ^207^ ^]^ and autopsy specimens^[^ ^208^, ^209^ ^]^ of AD. A*β* treatment induced depletion of GSH in cultured neurons.^[^ ^210^ ^]^ Recent evidence indicates that depletion of GSH destroyed redox homeostasis and is associated with the occurrence of ferroptosis in AD.^[^ ^211^, ^212^ ^]^

Moreover, a reduction of brain GPX4 level was observed in AD mice.^[^ ^213^ ^]^ Inhibition of GPX4 decreases the sensitivity of brain to ferroptosis in AD pathogenesis.^[^ ^63^ ^]^ Overexpression of GPX4 protects cortical neurons against A*β*‐induced cytotoxicity through suppressing LPO.^[^ ^214^ ^]^ Ablation of GPX4 can cause a neuronal loss in the hippocampal regions in neonatal^[^ ^23^ ^]^ and adult mice,^[^ ^215^ ^]^ accompanied by elevated activation of astrocytes,^[^ ^215^ ^]^ suggesting an suppressive function of GPX4 in neurodegenerative disorders. The conditional deletion in forebrain neurons of GPX4 in mice leads to cognitive impairment and hippocampal neurodegeneration.^[^ ^212^ ^]^ Moreover, ferroptosis inhibitors can reverse GPX4 ablation‐induced elevation of LPO, activation of ERK and augmentation of neuroinflammation, which are markers associated with ferroptosis, thereby ameliorating neurodegeneration.^[^ ^212^ ^]^ Overexpression of GPX4 inhibited neuronal loss and the production of lipid ROS in frontal cortex, accompanied by a reduced amyloid plaque formation in frontal cortex tissues and improved learning and memory abilities in 5×FAD (5×FAD/GPX4) mice.^[^ ^216^ ^]^ 5×FAD/GPX4 mice exhibited attenuated markers of ferroptosis, evidenced by decreased 4‐HNE, supporting the notion that ferroptosis is a key contributor to AD pathogenesis.^[^ ^216^ ^]^ A*β*_1‐42_ induced PC12 cell death via inducing VDAC1‐dependent ferroptosis, which was reversed by inhibition of VDAC1, thereby activating the AMPK/mTOR and Wnt/*β*‐catenin pathways.^[^ ^217^ ^]^ These findings strongly suggested that inhibition of the GSH/GPX4 axis is associated with AD pathology.

##### The Role of FSP1‐CoQH_2_ System in AD

Recent study has shown that the upregulation of FSP1 is involved in the inhibition of a ketogenic diet for chronic sleep deprivation‐induced AD.^[^ ^218^ ^]^ Ketogenic diet prevents the chronic sleep deprivation‐induced cognitive deficiency, amyloid deposition, and hyperphosphorylated tau protein. Ketogenic diet inhibits iron dyshomeostasis by down‐regulating the expression of TfR1 and DMT1 and up‐regulating the expression of FTH1 and FPN1. Ketogenic diet promoted the elevation of xCT/GPX4 axis, FSP1, and reduced MDA. Meanwhile, ketogenic diet activated Sirt1/Nrf2 signaling pathway in the hippocampus in SD‐exposed mice. These results suggested that KD could prevent chronic SD‐induced AD through inhibiting ferroptosis via Sirt1/Nrf2 signaling pathway, thereby upregulating GPX4 and FSP1.^[^ ^218^ ^]^

##### The Role of AD‐Related Regulator in AD

Mutations in presenilin 1 and 2 (PS1 and PS2) or the amyloid‐*β* (A*β*) precursor protein (APP), all of them are involved in the generation of A*β*, cause autosomal dominant familial AD (FAD). A recent study demonstrates that the potential for presenilin mutations to promote neurodegeneration irrespective of A*β*is through sensitizing multiple cell types to ferroptosis.^[^ ^219^ ^]^ Mutant PS1(mPS1) sensitizes cells to ferroptosis. mPS1 promotes the expression of GPX4 by quenching the membrane propagation of lethal hydroperoxyl radicals. Presenilin *γ*‐secretase activity cleaves Notch‐1 to signal LRP8 expression, which then controls GPX4 expression by regulating the supply of selenium into the cell since LRP8 is the uptake receptor for selenoprotein P. mPS1 disrupts selenium uptake, thus suppressing GPX4 expression. Presenilin mutations may promote neurodegeneration by promoting ferroptosis, which highlights a disease‐modifying concept for therapeutics.^[^ ^219^ ^]^ Allelic variation of APOE gene confers the greatest genetic risk for sporadic AD (SAD). Recent study reported that apoE activates the PI3K/AKT pathway to inhibit ferritinophagy, thus inhibiting iron‐dependent LPO.^[^ ^220^ ^]^ Postmortem study has revealed that there is a strong association of iron with pathologically confirmed clinical AD, particularly in those with the adverse APOE‐*ε*4 allele. APOE *ε*4 carriers increased susceptibility to ferroptosis in AD. APOE *ε*4 carriers express high levels of oxidation‐sensitive PUFAs that promote ferroptosis. The decreased level of apoE boost ferritinophagy and iron release from ferritin, which further increases LPO and ferroptosis.^[^ ^220^ ^]^

#### 4.1.3 Amyotrophic Lateral Sclerosis

Amyotrophic lateral sclerosis (ALS), also known as motor neuron disease (MND), is a devastating, progressive, and late‐onset neurodegenerative disease affecting the upper motor neurons in the motor cortex and lower motor neurons in the brainstem and spinal cord, leading to progressive muscle weakness and death from respiratory failure within 2–5 years of symptom onset.^[^ ^221^, ^222^, ^223^ ^]^ The neuropathological hallmark of ALS is the aggregation and accumulation of ubiquitinated proteinaceous inclusions in the motor neurons.90% of ALS cases are considered sporadic ALS (sALS) without correlation with family history, while about 10% of people with ALS show some form of family history, classifying their condition as familial ALS (fALS).^[^ ^223^ ^]^ More than 30 genes have been identified which are causative in or confer an increased risk of the development of ALS. The most common mutations occur in Cu/Zn superoxide dismutase 1(SOD1), TAR DNA‐binding protein 43 (TDP‐43), fused in sarcoma (FUS), and Chromosome 9 open reading frame 72(C9orf72), accounting for the disease in up to 70% of patients with fALS.^[^ ^221^ ^]^

##### Role of Iron and Ferroptosis in ALS

Brain iron metabolic deregulation and ferroptosis were associated with neurodegeneration in ALS in the studies in the last two decades. Increased levels or deposition of iron were found in the spinal cord of transgenic SOD1^G93A^ and SOD1^G37R^ mice^[^ ^224^, ^225^, ^226^ ^]^ and in motor cortex and substantia nigra of ALS patients.^[^ ^227^, ^228^ ^]^ Increased levels of ferritin were detected in the serum and in CSF of ALS patients,^[^ ^229^, ^230^, ^231^, ^232^, ^233^, ^234^ ^]^ which were associated with a poor clinical outcome.^[^ ^229^, ^230^, ^232^, ^233^, ^234^, ^235^ ^]^ An abnormal iron accumulation was observed in the tissues of SOD1^G93A^ mice already at a pre‐symptomatic stage of the disease.^[^ ^224^, ^236^ ^]^ Increased levels of iron were correlated with an increase of markers of LPO throughout the disease progression,^[^ ^236^ ^]^ suggesting that the progression of ALS symptoms was correlated with abbrant iron accumulation and increased LPO.

##### LPO and Ferroptosis in ALS

ROS production and lipid peroxidation have been shown to be a hallmark of pathology in ALS, where excessive levels of 4‐HNE have been found in the serum and cerebrospinal fluid in sALS patients.^[^ ^237^ ^]^ 4‐HNE levels were positively correlated with the disease stage.

##### The Role of Inhibition of GSH/GPX4 Axis in ALS

Another parameter that associates ALS to ferroptosis is GPX4. In neuronal inducible knockout of GPX4 mouse model, the conditional ablation of GPX4 led to a rapid development and progression of a motor phenotype, i.e., paralysis, severe muscle atrophy and, consequently, death.^[^ ^238^ ^]^ The dramatic motor neuron degeneration observed in the spinal cord of the mouse model was correlated with a significant decrease of GPX4 level and with an increase of 4‐HNE, the markers of ferroptosis.^[^ ^238^ ^]^ Chen et al. reported a compromised anti‐ferroptosis defense in SOD1^G93A^ mice and ALS patient samples, which revealed a significant decreased GPX4 activity and GSH levels in the spinal cord of SOD1^G93A^ animal models and in samples from sALS patients, that were correlated with an increase in lipid peroxidation products.^[^ ^239^ ^]^ It has been shown that the overexpression of GPX4 rescued motor neuron integrity and reduced the levels of lipid peroxidation in the spinal cord of the models.^[^ ^239^ ^]^ Furthermore, a delay in the disease onset, a slower progression of the motor symptoms, and an extension of the overall survival were observed.^[^ ^239^ ^]^ A most recent study showed that ferroptosis mediates selective motor neuron death in ALS.^[^ ^240^ ^]^ Depletion of GPX4 occurred in post‐mortem spinal cords of both sALS and fALS patients. GPX4 depletion was also an early and universal characteristic of spinal cords and brains of SOD1^G93A^, TDP‐43 and C9orf72 mouse models of ALS. GPX4 depletion resulted from an impaired Nrf2 pathway and GSH synthesis in mutant SOD1 mice.^[^ ^240^ ^]^ GPX4 overexpression improves lifespan, motor function and delays disease onset in SOD1^G93A^ mice, which was attributed to attenuated lipid peroxidation and motor neuron preservation.^[^ ^240^ ^]^

##### The Role of Inhibition of FSP1‐CoQH_2_ System Axis in ALS

The myeloperoxidase (MPO), a heme‐containing enzyme, converts hydrogen peroxide (H_2_O_2_) and Cl‐ to H_2_O to form hypochlorous acid (HOCl), a powerful oxidant of the reactive oxygen species (ROS) family.^[^ ^241^ ^]^ MPO/HOCl pathway was activated by hSOD1^G93A^ mutation in SOD1^G93A^ ALS NSC‐34 motor neuron models. Activation of the MPO/HOCl pathway occurred differently in motor neurons of the motor cortices, brain stems, and spinal cords in male and female SOD1^G93A^ transgenic mice. Inhibition of MPO improved the motor performance of SOD1^G93A^ transgenic mice.^[^ ^242^ ^]^ The activation of MPO/HOCl pathways facilitated ferroptosis through inhibiting the expressions of GPX4 and NQO1 and thus leading to irreversible lipid peroxidation.^[^ ^242^ ^]^ These results suggested that aggregation of mutant hSOD1 proteins led to activation of the MPO/HOCl pathway, triggering ferroptosis in ALS.^[^ ^242^ ^]^ Overexpressed FSP1 could ameliorate ferroptosis in SOD1^G93A^ ALS NSC‐34 motor neurons models, evidenced by suppressed the MDA levels. FSP1 overexpression increased cell viability in hSOD1^G93A^ cells. NQO1 expression was increased by FSP1 overexpression, which compensated for the inability of GPX4, by potential cooperating with NQO1 in hSOD1^G93A^ cells.^[^ ^242^ ^]^

##### The Role of Inhibition of GCH1‐BH_4_ System in ALS

Compared with WT, an obvious increased lipid peroxidation, content of LIP, and decreased GSH/GSSG was observed in hSOD1^G93A^ cells. Meanwhile, upregulated ALOX15 and GDF15 (respectively a driver and a marker of ferroptosis), down‐regulated GCH1 and GPX4, and unaltered FSP1 was observed in hSOD1^G93A^ cells. Mutant SOD1 promotes ferroptosis via generating TfR1‐imported excess free iron, decreasing GSH, upregulating ALOX15, and inactivating GCH1 and GPX4 in ALS.^[^ ^243^ ^]^ The study also showed that a highly conserved “cyclin‐like” protein, speedy/RINGO cell cycle regulator family member A (SPY1) resists ferroptosis by upregulating GCH1/BH_4_ and downregulating TfR1 in ALS.^[^ ^243^ ^]^

### 4.2 Acute Injury of Central Nervous System

#### 4.2.1 Ischemic Stroke

##### The Role of Iron Metabolism Dysregulation in Ischemic Stroke

**Figure 6:**
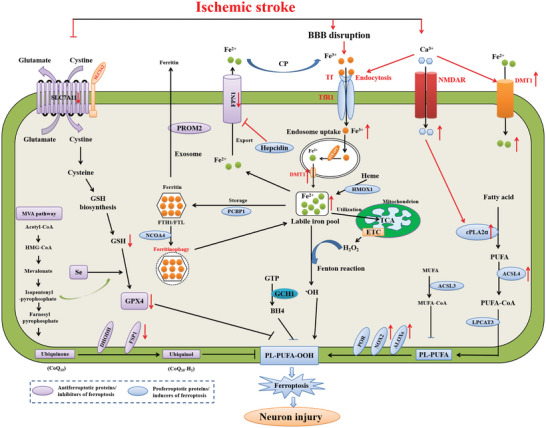
The molecular mechanisms of ferroptosis during cerebral ischemia. Following ischemic stroke, the stimulation of the NMDA receptor increases Ca^2+^, which activates cPLA2*α* to provide substrates for lipid peroxidation. NMDAR overactivation boosts neuronal iron uptake and produces ferroptosis. The dyshomeostasis of glutamatergic neurotransmission elevates extracellular glutamate levels, which inhibits cystine uptake and limits the biosynthesis of GSH. The reduction of soluble tau protein after cerebral ischemia prevents the dissociation of immature APP from the ER, abolishing the trafficking of APP to the neuronal surface, where APP interacts with FPN, allowing iron export from neurons. The absence of this interaction prevents iron from exiting neurons, leading to a toxic intracellular accumulation of iron and, ultimately, neuronal ferroptotic damage after ischemic stroke.

The dysregulation of iron metabolism and iron accumulation occurs in the brain after ischemic stroke^[^ ^244^ ^]^ (**Figure** **6**). The acidic environment in brain tissue following cerebral ischemia can inhibit the binding of iron to transferrin, leading to iron disassociation from transferrin.^[^ ^245^ ^]^ The neurons easily take up this unbound iron, causing intracellular iron elevation.^[^ ^246^ ^]^ In the middle cerebral artery occlusion (MCAO) ischemic stroke model, increased accumulation of iron in cells along the lesion border is detected at 72 h after stroke.

Ferritin protein is significantly increased in astrocytes and macrophages along the lesion border in the ischemic cortex.^[^ ^247^ ^]^ At 14 days, the iron is enriched predominately at macrophages of the entire ischemic lesion core, suggesting that astrocytes and macrophages play a role in regulating iron after cerebral ischemia. Ferritin,^[^ ^248^ ^]^ TFR1,^[^ ^249^, ^250^, ^251^, ^252^, ^253^ ^]^ and DMT1^[^ ^250^, ^252^ ^]^ which may contribute to the influx of iron, are also increased in ischemic stroke. Meanwhile, decreased iron efflux following ischemic stroke may also contribute to iron accumulation.^[^ ^247^, ^254^, ^255^, ^256^ ^]^

##### The Role of LPO in Ischemic Stroke

The brain is rich with PUFAs, making it more susceptible to LPO in ischemic conditions. The rapid ATP loss during cerebral ischemia leads to uncontrolled ion leakage across cell membranes, causing membrane depolarization and glutamate release.^[^ ^257^, ^258^, ^259^ ^]^ Excessive release of glutamate stimulates its receptors thereby resulting in the activation of phospholipases,^[^ ^257^, ^260^ ^]^ phospholipid hydrolysis, AA release,^[^ ^261^ ^]^ and the loss of repair capacity of GPX4 against lipid peroxide.^[^ ^262^, ^263^ ^]^ 12/15‐LOX that directly oxidizes lipid membranes containing PUFAs is a critical regulator of ferroptosis in neuronal damage after ischemic stroke. Elevated expression and activity of 12/15‐LOX were observed in the ischemic mouse brain, which colocalizes with MDA.^[^ ^264^, ^265^, ^266^ ^]^ The knockout of 12/15‐LOX protected neurons against cerebral ischemic injury.^[^ ^264^, ^267^ ^]^ ALOX15 knockdown increased the resistance to ferroptosis in neurons.^[^ ^268^ ^]^ The cytosolic phospholipase A2*α* (cPLA2*α*), a Ca^2+^‐dependent cytosolic enzyme, controls AA release from PUFAs.^[^ ^269^, ^270^ ^]^ Cerebral ischemia‐induced influx of Ca^2+^ activates cPLA2*α* to enhance LPO. cPLA2*α* is increased after cerebral ischemia, and its knockdown can significantly decrease LPO and brain injury.^[^ ^260^, ^271^, ^272^, ^273^ ^]^ Ischemic stroke upregulate ACSL4.^[^ ^251^, ^266^, ^274^, ^275^, ^276^, ^277^ ^]^ Overexpression of ACSL4 aggravates ischemic brain damage, while silencing ACSL4 protects mouse brain against ischemia‐induced injury through inhibiting ferroptosis.^[^ ^274^ ^]^

##### The Role of Inhibition of GSH/GPX4 Axis in Ischemic Stroke

The level of GSH is significantly reduced in in vitro and in vivo ischemic models.^[^ ^253^, ^276^, ^277^, ^278^, ^279^, ^280^ ^]^ The levels of SLC7A11^[^ ^250^, ^252^, ^253^, ^281^, ^282^, ^283^ ^]^ and GPX4^[^ ^249^, ^250^, ^251^, ^252^, ^253^, ^266^, ^275^, ^276^, ^277^, ^279^, ^280^, ^281^, ^282^, ^283^, ^284^ ^]^ were also found to be significantly decreased, while those of lipid peroxide are significantly increased in in vitro and in vivo ischemic models. In ischemic stroke, GSH supplement confers a therapeutic effect to suppress ischemic stroke‐induced brain injury.^[^ ^285^ ^]^ GSH synthesis and ferroptosis were also regulated by intracellular glutamate, with intracellular glutamate intake reduced after ischemic stroke, leading to an increased extracellular release,^[^ ^286^ ^]^ thereby inhibiting the system Xc^−^ and trigger ferroptosis.^[^ ^287^, ^288^ ^]^ The rapid energy loss during cerebral ischemia results in glutamate release.^[^ ^257^, ^258^, ^259^ ^]^ Excessive release of glutamate may lead to the loss of repair capacity of GPX4 against lipid peroxide.^[^ ^262^, ^263^ ^]^ Thus, increased extracellular glutamate after ischemic stroke may also trigger ferroptosis.

#### 4.2.2 Spontaneous Intracerebral Hemorrhage

**Figure 7:**
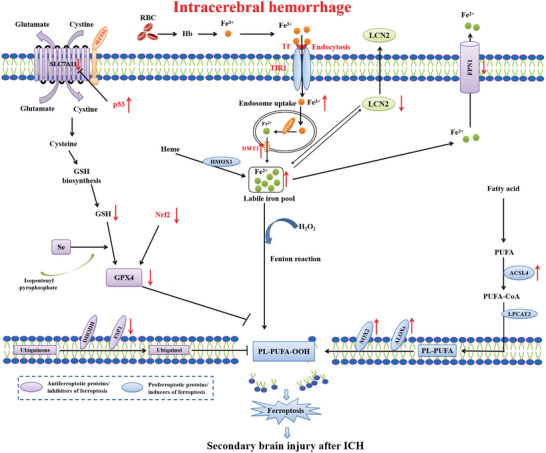
Mechanisms governing ferroptosis in ICH. Following a hemorrhagic stroke, iron derived from hemoglobin and/or heme enters neurons and produces massive lipid peroxidation. The increased permeability of the blood‐brain barrier (BBB) causes a variety of components rich in Fe^3+^ in the bloodstream to infiltrate into the brain parenchyma. Fe^3+^‐TF then binds to TfR1 on the surface of brain cell membranes, enters cells through endocytosis. Fe^3+^ is reduced to Fe^2+^, and transported to the cytoplasm through DMT1. Fe^2+^ then initiates the Fenton reaction to form reactive oxygen species (ROS) and affects the catalytic activity of lipoxygenase (LOX). Elevated extracellular glutamate leads to an inhibition of system XC^−^, depletion of GSH, and diminution in GPX4 activity. Meanwhile, glutamate binds to and activates NMDAR, which further exacerbates iron uptake. Regarding lipid metabolism, ACSL4 is upregulated and facilitated the production of PUFA‐CoA and PL‐PUFAs which is then catalyzed by LOX to PL‐PUFA‐OOH. These components together lead to ferroptosis, account for the secondary brain injury after ICH.

Spontaneous intracerebral hemorrhage (ICH) is an acute subtype of acute cerebral stroke and accounts for 80% of hemorrhagic stroke and 10–15% of all types of strokes.^[^ ^289^ ^]^ ICH causes two types of injury to the brain. The first is the primary brain injury, which is caused by the hematoma compressing the surrounding brain tissues. The second injury is the secondary brain injury after ICH (SBI‐ICH), which is caused by blood components, such as hemoglobin (Hb), iron, and other neurotoxic substances released by the hematoma, all of which contribute to oxidative stress and neuroinflammation.^[^ ^290^, ^291^ ^]^ Both the primary injury and SBI‐ICH lead to significant loss of neurological functions. Previous studies have shown that ferroptosis occurs in the ICH model in mice, and contributes to ICH‐induced neuronal death.^[^ ^292^ ^]^ A growing experimental evidence implicates that ferroptosis is involved in the pathogenesis of ICH^[^ ^290^, ^291^ ^]^ (**Figure** **7**).

##### The Role of Iron Metabolism Dysregulation in ICH

Iron accumulation occurs and causes brain damage after ICH.^[^ ^293^ ^]^ DMT1 and Fpn increase in ICH rats, both of which are positively correlated with Fe^2+^.^[^ ^294^ ^]^ Certain lines of evidence suggest that dysregulation of proteins involving in iron influx and efflux, and mobilization of iron stored in ferritin (i.e., ferritin, TF, and TfR levels and HO‐1), were significantly increased in the brain after ICH, leading to increased intracellular redox‐active iron.^[^ ^295^ ^]^ Following ICH, lysed red blood cells released Hb, which can be engulfed by activated microglia and macrophages in the perihematomal zone and release ferrous/ferric iron, thereby inducing the formation of lethal ROS and LPO.^[^ ^296^, ^297^, ^298^, ^299^ ^]^ Subsequently, excessive ferrous iron accumulates in neurons is transported out of microglia via the transferrin (Tf)‐Tf receptor system and reacts with H_2_O_2_ to form hydroxyl radicals (•OH) with a highly toxic effect via the Fenton reaction.^[^ ^300^ ^]^ Heme oxygenase (HO) is the initial and rate‐limiting enzyme of heme catabolism that catalyzes the oxidation of heme to Fe^2+^, and the expression of Heme oxygenase‐1 (HO‐1) is rapidly induced following ICH.^[^ ^301^, ^302^ ^]^ HO‐1 knockout mice show smaller infarct volumes after ICH,^[^ ^303^ ^]^ indicating a harmful role played by HO‐1 in ICH. It was revealed that overexpression of *IRP2* mRNA after ICH,^[^ ^292^ ^]^ and the ablation of *IRP2* in neurons can possess neuroprotection against the toxicity of Hb after ICH.^[^ ^304^ ^]^ The attenuation of hemoglobin‐induced toxicity was also observed in cortical cell cultures from *IRP2* knockout mice.^[^ ^305^ ^]^ The molecular mechanism underlying the neuroprotection in *IRP2* knockout mice is likely related to sequester the excess hemorrhage‐induced iron by increasing the expression of ferritin. Recent studies using in vitro and in vivo models have revealed that iron elevation and deposition were increased after ICH.^[^ ^306^, ^307^, ^308^, ^309^, ^310^, ^311^, ^312^ ^]^ In hemin‐treated N2A and SK‐N‐SH neuronal cells, hemin induced ferroptosis accompanied by an increment of global level of trimethylation in histone 3 lysine 9 (H3K9me3) and its methyltransferase Suv39h1. H3K9me3 was enriched at the promoter region and transferrin receptor gene 1 (Tfr1) and repressed its expression upon hemin stimulation. Inhibition of H3K9me3 or knockdown of Suv39h1 aggravated ferroptosis by upregulating Tfr1 expression. Furthermore, Suv39h1‐H3K9me3 mediated repression of Tfr1 contributes to the progression of ICH in mice. These data suggest that H3K9 trimethylation dictates neuronal ferroptosis through repressing Tfr1 post ICH.^[^ ^313^ ^]^

##### The Role of LPO in ICH

ICH‐associated ROS can cause cell damage through ferroptosis‐like LPO.^[^ ^314^ ^]^ The ICH promotes heme incorporation into the plasma membrane, thereby increasing the sensitivity to the exogenous H_2_O_2_ to facilitate LPO.^[^ ^300^, ^315^ ^]^ It was shown that heme significantly increased the contents of MDA and 4‐HNE and significantly depleted glutathione in primary hippocampal neurons.^[^ ^316^ ^]^ The content of 4‐HNE and MDA were increased in brain tissue around hematoma 7 days after ICH in ICH model rats.^[^ ^316^ ^]^ The content of MDA was significantly increased after ICH in mice.^[^ ^311^ ^]^ This observation was corroborated by other studies.^[^ ^306^, ^307^, ^308^, ^309^, ^312^, ^317^, ^318^ ^]^ Previous studies have demonstrated that specific regulators of ferroptosis, including CDGSH iron sulfur domain 2 (CISD2),^[^ ^319^ ^]^ NOX4,^[^ ^320^ ^]^ SRY‐box transcription factor 10 (SOX10),^[^ ^321^ ^]^ and forkhead box O3 (FOXO3)^[^ ^322^ ^]^ play crucial roles in regulating ferroptosis in secondary brain injury after ICH. Increased CISD2 alleviates brain injury by inhibiting lipid peroxidation and ferroptosis via AKT/mTOR in mice.^[^ ^319^ ^]^ The transcription factor SOX10 inhibits ferroptosis of hippocampal neurons after ICH through increasing miR‐29a‐3p expression, resulting in suppression of ACSL4 transcription.^[^ ^321^ ^]^ Silencing FOXO3 ameliorates post‐ICH brain damage through inhibiting neuronal ferroptosis via down‐regulating NOX4 transcription levels.^[^ ^322^ ^]^

##### The role of Inhibition of GSH/GPX4 Axis in ICH

The depletion of GSH and the reduction of GPX4 induced ferroptosis after ICH. GSH was decreased following ICH^[^ ^307^, ^308^, ^316^, ^323^ ^]^ and caused brain edema and neural injury, which were reversed by GSH supplement.^[^ ^324^ ^]^ The downregulation of glutathione reductase may account for the depletion of GSH after ICH.^[^ ^309^ ^]^ previous investigations have shown that GPX4 levels were markedly reduced in neurons after ICH,^[^ ^263^, ^307^, ^310^, ^311^, ^312^, ^316^, ^317^, ^318^, ^325^, ^326^ ^]^ the inhibition of GPX4 could exacerbate SBI‐ICH, while the overexpression of GPX4 ameliorates ferroptosis‐mediated hemorrhagic brain injury in rats.^[^ ^327^ ^]^ The expression of SLC7A11 was markedly reduced in neurons after ICH.^[^ ^311^, ^318^, ^325^ ^]^ The FOXO3 has been reported to play crucial roles in regulating ferroptosis in secondary brain injury after ICH through repressing GPX4.^[^ ^322^ ^]^ Excitatory amino acid transporter 3 (EAAT3) that mediates mature neurons take up astrocyte‐derived cysteine and plays an important role in neuronal resistance to ferroptosis through maintaining the activity of the GSH‐GPX4 antioxidant pathway and scavenging lipid peroxides.^[^ ^328^ ^]^ NOX4‐mediated peroxidation and Tf/TfR‐mediated iron overload exacerbate neuronal ferroptosis in a rat model of ICH.^[^ ^320^ ^]^ In addition, astrocyte‐derived glutamine synthesis was increased in ICH rats, while neuronal cysteine uptake was diminished, evidenced by downregulation of EAAT3 and GPX4 expression. Inhibiting NOX4 and iron chelation partially restored neuronal levels of EAAT3 and GPX4 expression and inhibited neuronal ferroptosis, suggesting that inhibition of these pathological signals can protect the hemorrhagic brain.^[^ ^320^ ^]^ Increased methyltransferase‐like 3, a N_6_‐methyladenosine (m^6^A) methyltransferases (“writers”) promoted secondary brain injury after ICH by inhibiting GPX4 expression in an m^6^A‐dependent manner.^[^ ^329^ ^]^ These results implied that m^6^A‐dependent epigenetic modification of GPX4 is involved in the genesis of secondary brain injury after ICH.

#### 4.2.3 Subarachnoid Hemorrhage

**Figure 8:**
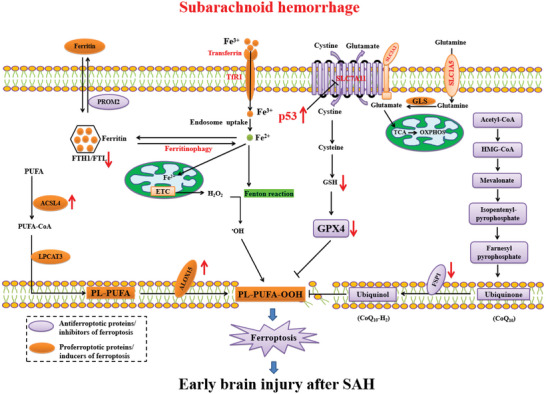
Mechanisms governing ferroptosis in SAH. Following SAH, the iron homeostasis is disrupted, intracellular LPO elevated and expression of GPX4 decreased, which induces ferroptosis, thereby leading to early brain injury after SAH. The iron metabolism is dysregulated, increased LPO and the inhibition of GSH/GPX4 were observed in SAH. ACSL4 was upregulated and FSP1 was downregulated in brain tissues after SAH. Elevated iron causes brain damage after SAH, which maybe be related to dysregulation of hepcidin.

Subarachnoid hemorrhage (SAH), an acute hemorrhagic stroke commonly caused by ruptured intracranial aneurysms. SAH is characterized by the complexity of pathophysiological responses following extravasation of blood from cerebral circulation, and carries high mortality and morbidity.^[^ ^330^ ^]^ Early brain injury after SAH (EBI‐SAH) plays an important role in the poor prognosis of SAH.^[^ ^331^ ^]^ Although the exact molecular mechanisms of EBI‐SAH are being elucidated, it is widely accepted that increased oxidative stress, blood‐brain barrier disruption, neuroinflammation, and cell death within the brain contribute to brain injury.^[^ ^331^, ^332^ ^]^ A previous study has shown that ferroptosis occurs in the EBI‐SAH, and contributes to neuronal death after SAH,^[^ ^333^ ^]^ accumulating evidence show ferroptosis is involved in the pathogenesis of EBI‐SAH (**Figure** **8**). Using both in vivo and in vitro models, it was shown that iron metabolism dysregulation, increased LPO and the inhibition of GSH/GPX4 were observed in in vivo and in vitro models with SAH.^[^ ^333^ ^]^

##### The Role of Iron Metabolism Dysregulation in SAH

Elevated iron causes brain damage after SAH,^[^ ^334^, ^335^ ^]^ which is related to dysregulation of hepcidin. Pharmacological upregulation of hepcidin increased the expression of DMT1, decreased the expression of FPN1, and aggravated ferroptosis and EBI‐SAH, indicating that hepcidin played a role in regulating iron metabolism and contribute to ferroptosis via activation of DMT1 signaling in rats with SAH.^[^ ^336^ ^]^

A recent study showed that ferritinophagy is involved in the pathogenesis of EBI‐SAH through inducing ferroptosis.^[^ ^337^ ^]^ As a type of autophagy, ferritinophagy mediated by nuclear receptor activator 4 (NCOA4) plays a role in inducing ferroptosis by regulating iron homeostasis and producing ROS in cells.^[^ ^338^, ^339^, ^340^ ^]^ NCOA4 acts as a selective autophagy receptor and binds to FTH1 of ferritin to mediate the transport of intracellular ferritin to autophagy lysosomes and finally releases free iron, which increases the content of available iron in cells.^[^ ^341^ ^]^ SAH can disrupt iron homeostasis, elevated intracellular LPO, and decreased expression of GPX4 and FTH1. The autophagy inhibition by ATG5 gene knockout can reduce the intracellular iron level and LPO, increase the expression of GPX4, thereby alleviating SAH‐induced cell death, suggesting that SAH triggers neuronal ferritinophagy‐dependent ferroptosis and regulating iron homeostasis through ferritinophagy, which provides clues for the prevention of EBI‐SAH.^[^ ^337^ ^]^ Transferrin infiltration was increased in the brain parenchyma 24 h after SAH, which was positively correlated with neuronal ferroptosis. Overexpression of aquaporin 4 in the mouse brain can effectively improve post‐SAH neuronal ferroptosis and brain injury through inhibiting transferrin infiltration into the brain parenchyma.^[^ ^342^ ^]^

##### The Role of LPO in SAH

ACSL4 level in brain tissue increased significantly after SAH. Inhibiting ACSL4 alleviated EBI‐SAH, evidenced by decreased inflammation, blood‐brain barrier (BBB) impairment, oxidative stress, brain edema, and behavioral and cognitive deficits, and increased the number of surviving neurons, indicating that ACSL4 exacerbated SAH‐induced EBI by mediating ferroptosis.^[^ ^343^ ^]^

##### The Role of Inhibition of GSH/GPX4 Axis in SAH

The depletion of GSH^[^ ^335^, ^344^, ^345^ ^]^ and the reduction of GPX4^[^ ^335^, ^345^, ^346^, ^347^, ^348^ ^]^ induced ferroptosis after SAH, leading to EBI‐SAH. The overexpression of GPX4 ameliorates ferroptosis‐mediated EBI‐SAH, evidenced by reduced LPO and cell death in both in vitro and in vivo experimental SAH models, and decreased brain edema and neurological deficits after SAH.^[^ ^349^ ^]^ However, the role of ferroptosis in EBI‐SAH remains an open conundrum for future investigation.

## 5 Pharmacological Inhibition of Ferroptosis to Treat NDs and Strokes

It was shown that Fer‐1 prevents glutamate induced ferroptosis cell death in postnatal rat brain, suggesting that the inhibition of ferroptosis could be exploited to preserve neuronal cells and protect organisms from specific oxidative, iron‐dependent neurodegeneration, such as AD, PD, and strokes.^[^ ^1^ ^]^ The existing evidence suggests a strong correlation between ferroptosis and neurodegenerative diseases and stroke through a shared mechanism involving dysregulation of iron metabolism, induction of LPO, and inhibition of GSH/GPX4 axis. Thus, inhibition of ferroptosis could be a promising target for treatment of neurodegenerative diseases and stroke. Since the discovery of the involvement of ferroptosis in pathogenesis of neurological diseases, scientists have claimed an approach of targeting anti‐ferroptosis to treat these diseases. A growing number of drugs have been uncovered to exert their therapeutic efficacy by inhibiting ferroptosis. During the past decade, many experimental compounds and clinical drugs have been shown to inhibit ferroptosis by preclinical and clinical studies to achieve therapeutic purposes. Mounting evidence indicates that pharmacological inhibition of ferroptosis exert neuroprotection in in vitro or in vivo disease models in PD (**Table** **1**), AD (**Table** **2**), ALS (**Table** **3**), IS (**Table** **4**), and ICH (**Table** **5**), and SAH (**Table** **6**).

**Table 1:** Emerging compounds targeting key regulators of ferroptosis to attenuate PD (ACSL4, long‐chain acyl‐CoA synthetase 4; DFO, deferoxamine; DMT1, iron importer divalent metal transporter 1; FPN1, iron efflux transporter; FSP1, ferroptosis suppressor protein 1; FTH1, Ferritin Heavy Chain 1; GW501516, a specific PPAR*δ* agonist; IRP1, iron regulatory protein 1; NBP,DL‐3‐n‐butylphthalide; NQO1,NAD(P)H dehydrogenase[quinone]‐1; TFR2, iron uptake transporter)

Compounds	Experimental model	Findings	Mode of action	Ref
(‐)‐Clausen amide	MPTP/C57BL/6J mice	↓Behavioral defects;↓injured dopaminergic neurons;↓nuclear translocation of ALOX5, which was essential for catalyzing the production of toxic lipids 5‐HETE;↑GPX4;↓TfR1;↓MDA	↑GPX4	[403]
Quercetin	MPTP/C57BL/6 mice	↓Behavioral disorders;↓dopaminergic neurons death;↑Nrf2;↑SLC7A11;↑GPX4	↑Nrf2/xCT/GPX4 axis	[350]
Quercetin	MPP^+^/M17 cell or PC12	↑Cell viability;↑GPX4;↓Lipid ROS;↑Nrf2;↑SLC7A11;↑FTH;↑GSH, and SOD;↓MDA	↑Nrf2/xCT/GPX4 axis	[350]
Dl‐3‐n‐butylphthalide	MPP^+^/N2A Cells	↑Cell viability;↑p53	↑p53	[351]
*β*‐Hydroxybutyric acid	MPTP/C57BL/6 mice	↑Cell viability;↓oxidative stress;↓TH↓ a‐syn;↓ACSL4;↑GPX4;↑FTH1;↑GSH;↓MDA↓Fe^2+^ content	↑GPX4 axis;↓Fe^2+^;↓ACSL4	[352]
*β*‐Hydroxybutyric acid	MPP^+^/SN4741	↑Cell viability;↓oxidative stress;↓TH↓; a‐syn↓ACSL4;↑GPX4;↑FTH1;↑GSH;↓MDA↓Fe^2+^ content;↑ZFP36	↑GPX4 axis;↓Fe^2+^;↓ACSL4	[352]
Quercetin	MPP^+^/PC12	↑Cell viability;↓ROS;↑MMP;↑intracellular ATP levels;↑GSH;↑SLC7A11 and GPx4; ↓ACSL4; ↑transferrin;↑Nrf2	↑Nrf2/xCT/GPX4 axis	[350]
Quercetin	MPTP/mice	↓MPTP‐induced motor deficits;↓loss of neuron in SN;↑GSH, and SOD;↓MDA;↑Nrf2	↑Nrf2/xCT/GPX4 axis	[350]
Dl‐3‐n‐butylphthalide	Rotenone/rat	↓Rotenone‐induced motor disturbance;↓ loss of dopaminergic neurons and aggregation of *α*‐synuclein;↓ iron deposition in the SN and iron content in serum; ↓TfR;↓ Ft‐L;↑Fpn1; ↓ MDA;↑GPX4 and ↑SLC7A11;↑GSH;↓ ROS	↑xCT/GPX4 axis	[109]
Thonningianin A	6‐OHDA/zebrafish	↑ Total swimming distance of zebrafish larvae; ↓aggregation of *α*‐syn;↓ MDA, iron;↑GSH	↑Nrf2/xCT/GPX4 axis	[112]
Thonningianin A	6‐OHDA/SH‐SY5Y	↑Cell viability;↓Lipid ROS;↓ MDA, iron;↑GSH;↓aggregation of *α*‐syn;↑GPX4; ↓ACSL4; ↑transferrin;↑Nrf2/HO‐1	↑Nrf2/xCT/GPX4 axis	[112]
GW501516	6‐OHDA/SH‐SY5Y	↑Cell viability;↓ROS;↓ MDA;↓Lipid ROS;↓iron;↓DMT1 protein and mRNA;↑FPN1 protein;↓IRP1	↓Fe^2+^	[111]
Hinokitiol	6‐OHDA/PC12	↑Cell viability; DFO treatment increases the cell viability;↓ROS;↓ MDA;↓Lipid ROS;↑Fpn1;↓FTH;↑TfR1	↓Fe^2+^	[113]
Paeoniflorin	MPP^+^/primary neuron culture	↑Cell viability; ↑GSH;↑GPx4;↓Lipid ROS;↑Akt/Nrf2	↑Nrf2/GPX4 axis	[125]
*α*‐Lipoic acid	MPP^+^/PC12	↑Cell viability;↓ MDA, 4‐HNE, iron, and ROS;↑GSH;↑SLC7A11 and GPx4;↑PI3K/Akt/Nrf2	↑Nrf2/xCT/GPX4 axis	[114]
DFO	MPP^+^/PC12	↑TH;↓ ROS;↓ DMT1; ↓TfR1; ↓FPN; ↓ACSL4; ↑GPX4;↑FTH1	↓ ACSL4;↑GPX4 axis	[353]
SK4/DFO	MPP^+^/LUHMES cells	↑Cell viability;↓Oxidative stress; ↓Fe^2+^ content;	↓Fe^2+^	[354]
Clioquinol	MPTP/monkey	↓Motor and non‐motor deficits;↓iron content and ROS level in the SN; ↓apoptosis;↑AKT/mTOR;↓p53 medicated cell death;↓TRF2 mRNA;↑FPN1 mRNA;↑GSH in the SN;↑SOD, GSH, and MDA levels in serum;↓4‐HNE in the SN	↑Nrf2/HO‐1 pathway	[115]
Apoferritin	MPTP/mice	↓MPTP‐induced motor deficits;↓iron aggregation;↓DMT1; ↓ ACSL4;↓ FSP1	↓ ACSL4	[126]
Ferrostatin‐1	6‐OHDA/zebrafish	↓ROS; ↓MDA;↓Iron content↓;↑GSH	↓LPO;↓Fe^2+^	[110]
Ferrostatin‐1	6‐OHDA/SH‐SY5Y	↓Lipid ROS; ↓MDA;↓ ACSL4; ↑GPX4; ↓Iron content;↓*α*‐syn;↓ ↑GSH	↓ ACSL4;↑GPX4 axis	[110]
SK4/DFO	6‐OHDA/LUHMES cells	↑Cell viability;↓Oxidative stress; ↓Fe^2+^ content	↓Fe^2+^	[354]
Ferrostatin‐1	Rotenone/SH‐SY5Y	↓ROS/RNS;↓PARP‐1 Cleavage; ↓ER‐mediated Stress Response;↓*α*‐syn Aggregation	‐	[355]
Idebenone	Rotenone/rat	↓LPO;↑GPX4;↓oxidative stress motor impairment;↑tyrosine hydroxylase‐positive neuron survival;↑NQO1	↑GPX4	[356]

**Table 2:** Emerging compounds targeting key regulators of ferroptosis to attenuate AD (7,8‐DHF, 7,8‐Dihydroxyflavone; ACSL4, long‐chain acyl‐CoA synthetase 4; DHMDC, 2′,6′‐dihydroxy‐4′‐methoxy dihydrochalcone; DMT1, iron importer divalent metal transporter 1; FPN1, iron efflux transporter; FSP1, ferroptosis suppressor protein 1; FTH1, Ferritin Heavy Chain 1; N2L, a novel lipoic acid‐niacin dimer; OABL, 1,6‐O,O‐diacetylbritannilactone; TFR2, iron uptake transporter; TSG, Tetrahydroxy stilbene glycoside; *γ*‐GC,*γ*‐glutamylcysteine.)

Compounds	Experimental model	Findings	Mode of action	Refs.
Salidroside	SAMP8 mice	↓Cognitive impairment;↓A*β* plaques;↓neuronal damage;↓infiltration of CD8+T cells, oxidative stress, and inflammatory cytokines;↓iron deposition;↓TFR1;↓ACSL4;↑SLC7A11, and GPX4;↑Nrf2	Nrf2/GPX4	[357]
Edaravone	A*β*_1−42_/HT22 cells	↓Apoptosis of HT22 cells;↓TNF‐*α*, IL‐1*β* and IL‐6;↓TLR4/NF‐*κ*B/NLRP3;↓ferroptosis;↓lipid peroxidation	↓ LPO	[359]
Senegenin	A*β*_25–35_/PC12 cell	↑Cell viability;↓ ROS;↑GPX4;↓MDA;↓ACSL4 and PEBP1	↑GPX4	[360]
Salidroside	A*β*_1−42_/mice	↓Cognitive dysfunction;↓ultrastructural changes in mitochondria;↑GPX4, HO1, and NQO1;↓PTGS2	↑GPX4	[358]
Salidroside	Glutamate/HT22 cells	↑Cell viability and the level of MMP;↓ultrastructural changes in mitochondria;↓Fe^2+^ content;↓MDA;↑SOD and the ratio of GSH/GSSG;↓ ROS;↑GPX4 and SLC7A11; ↑Nrf2	↑Nrf2/GPX4	[358]
TSG	APP/PS1 mice	↓Oxidative stress;↓LPO;↓DMT1, ACSL4 and NCOA4;↑SOD, and the expression of FTH1, CD98 and xCT	↓ LPO;↑GPX4;↑Nrf2	[361]
Ginkgolide B	SAMP8 mice	↓Cognitive dysfunction;↑GPX4; ↑FTH1; ↑Nrf2;↓TFR1; ↓NCOA4;↑SOD and GSH;↓MDA and ROS	↓LPO;↑GPX4; ↑Nrf2	[362]
Forsythoside A	APP/PS1 double transgenic AD mice	↓Cognitive dysfunction;↓neuroinflammation;↓A*β*_1‐42_ deposition and phosphorylated tau protein in the hippocampus;↓TfR1 and DMT1;↑FTH, and FTL;↑p‐GSK‐3*β*, GPX4, Nrf2;↓ALOX5	↑Nrf2/GPX4	[363]
Forsythoside A	A*β*_1‐42_/N2a cells	↑Cell viability;↓dissipation of MMP;↓MDA	↓LPO	[363]
Forsythoside A	Erastin/HT22 cells	↓Ferroptosis‐related inflammation	↓Neuroinflammation	[363]
Forsythoside A	LPS/BV2 cells	↓NO, IL‐1*β*, and IL‐6	↓Neuroinflammation	[363]
Deferoxamine	Aluminum maltolate /rat	↓Cognitive dysfunction;↓MDA and ROS;↑GSH	↓ LPO	[364]
Insamgobonhwan	RSL3/HT22 Cells	↑Cell viability;↓ lipid ROS;↑GPX4;↓COX2;↓activation of ERK and JNK	↑GPX4	[365]
Insamgobonhwan	A*β*_25–35_/mice	↓MDA in hippo	↓ LPO	[365]
*γ*‐GC	APP/PS1 mice	↑Spatial memory;↓LPO, protein carbonyls;↑GSH and GSH/GSSG ratio;↑GPX4;↑SOD	↓ LPO;↑GPX4	[366]
N2L	RSL3/HT22 cells	↓LPO and ROS; ↑GPX4;↓ACSL4 and COX2;↑FTH1	↓LPO; ↑GPX4; ↑Nrf2	[367]
Eriodictyol	APP/PS1 mice	↓ Cognitive deficits;↓A*β* aggregation and the phosphorylated level Tau in the brain;↑GPX4; ↑Fpn1;↓FTH1; ↓TfR1	↓LPO;↑GPX4	[368]
Selenium (Se)	3× Tg‐AD mice	Improved cognitive impairment and AD‐related pathological symptoms in mice	↑GPX4	[369]
Eriodictyol	A*β*_1‐42_ oligomer/HT‐22 cells	↓Fe^2+^ content;↑GPX4;↑Fpn1;↓FTH1;↓TfR1;↓ROS;↓MDA	↓LPO;↑GPX4;↓Iron	[368]
Clioquinol	‐	↓Tau deficiency impaired iron export	Iron	[371]
GW7647	APP/PS1 mice	↓A*β* burden;↓cognitive defect;↓LPO;↓Fe^2+^ content	Iron;↓LPO	[372]
*α*‐Lipoic acid	P301S Tau transgenic mice	↓Iron overload, LPO, and inflammation;↑Fpn1;↓TfR1; ↑GPX4;↑SOD1;↑xCT	↓Iron;↑GPX4;↓LPO	[373]
OABL	5×FAD Mice	↓impairments in cognitive function;↓A*β* plaques, the A*β* expression, the phosphorylation of Tau protein, and the expression of BACE1 in AD mice brain;↓MDA;↑GSH;	↓ LPO	[374]
CMS121	APPswe/PS1ΔE9‐transgenic mice	↓Cognitive dysfunction;↓4HNE; ↓15LOX2	↓LPO	[375]
Ferrostatin‐1(Fer‐1)	A*β*/primary neurons	Fer‐1/Lip‐1 effectively ameliorate A*β* induced neuronal death and memory loss	↓LPO	[173]
Liproxstatin‐1(Lip‐1)	Mouse mode	Fer‐1/Lip‐1 effectively ameliorate A*β* induced neuronal death and memory loss	↓LPO	[173]
Coenzyme Q10 (CoQ10)	APP/PS1 mice	Improved AD‐type behavioral and pathological symptoms;↓circulating amyloid‐*β* (A*β*) peptide;↓A*β* plaque formation	↓LPO	[376]
7,8‐DHF	STZ/rats	↑GSH, catalase, SOD, GPX;↓LPO	↓LPO	[377]
DHMDC	STZ/mice	↑GSH activity;↓LPO;↓oxidative stress	↓LPO	[378]
Kojic acid	A*β*_1‐42_/mice	↓A*β* and BACE‐1;↑ Nrf2 and HO‐1;↓LPO and ROS	↓LPO	[379]
Crocin	A*β*_1‐42_/rats	↓LPO and ROS	↓LPO	[380]
Celecoxib	A*β*/SH‐SY5Y	↓LPO and ROS;↑ HO‐1	↓LPO	[381]
Allicin	ALCl_3_/rats	↓LPO and ROS;↓LPO and ROS;↑ GSH	↓LPO	[382]
Centella asiatica	ALCl_3_/rats	↓LPO and ROS;↑ SOD	↓LPO	[383]
Ellagic acid	ALCl_3_/rats	↓LPO;↑ catalase;↑GSH	↓LPO	[384]
Selenium	STZ/rat	↓LPO;↑ catalase, ↑GSH;↑ SOD	↓LPO	[370]

**Table 3:** Emerging compounds targeting key regulators of ferroptosis to attenuate ALS

Compounds	Experimental model	Findings	Mode of action	Refs.
Fer‐1	NSC‐34 cells expressing mutant SOD1^G93A^	↑Cell viability	↓ LPO	[239]
Liproxstatin‐1	NSC‐34 cells expressing mutant SOD1^G93A^	↑Cell viability	↓ LPO	[239]
Vitamin E	GPX4NIKO mice	Delayed the onset of paralysis and death induced by GPX4 ablation	↑GPX4	[238]
Vitamin E	RSL3/iPS cell‐derived motor neuron	↓Cell death	‐	[385]
Deferoxamine	RSL3/iPS cell‐derived motor neuron	↓Cell death	‐	[385]
Salicylaldehyde isonicotinoyl hydrazone	SOD1^G37R^ transgenic mice	↓Iron accumulation and motor neuron degeneration; delayed the disease onset;↓progression of motor symptoms;↑lifespan of mice	↓ LPO;↑GPX4;↑Nrf2	[224]
2‐(2‐Hydroxyphenyl)‐benzoxazole	SOD1^G37R^ transgenic mice	↓SOD1 protein aggregation in the spinal cord;↓iron accumulation and lipid peroxidation markers in both spinal cord and low‐fat muscles;↓rates of denervation and muscle atrophy; delay in the disease onset	↓LPO;↑GPX4; ↑Nrf2	[386]
VAR10303	SOD1^G37R^ transgenic mice	↓MN degeneration in the spinal cord;↓ferritin and the denervation/atrophy markers in GNS muscle	↑Nrf2/GPX4	[387]
Deferiprone	SOD1^G37R^ transgenic mice	↑Survival and physical condition;↓iron accumulation in the spinal cord;↓muscle denervation; ALS patients treated with DFP present a significantly slower decrease in ALSFRS‐R scores and reduced iron and oxidative markers	↓LPO	[388]
Deferiprone	RSL3/NSC34 cells stably transfecting hSOD1^G93A^	↑Cell viability;↓Lipid ROS		[243]
Fer‐1	RSL3/NSC34 cells stably transfecting hSOD1^G93A^	↑Cell viability;↓Lipid ROS		[243]

**Table 4:** Emerging compounds targeting key regulators of ferroptosis to attenuate ischemic stroke (ACSL4, long‐chain acyl‐CoA synthetase 4; ALOX5,5‐lipoxygenase; CCA, common carotid arteries; DMT1, iron importer divalent metal transporter 1; Fn, iron transporters ferritin; FLC, ferritin light chain; FPN1, ferroportin 1(iron efflux transporter); FSP1, ferroptosis suppressor protein 1; FTH1, Ferritin Heavy Chain 1; GSH, glutathione; GSSG, oxidized glutathione; HYSA, hydroxysafflor yellow A; LTCC,L‐type calcium channel; MDA, malondialdehyde; MPO, myeloperoxidase; NQO1, NAD(P)H dehydrogenase[quinone]‐1; OGD/R, oxygen‐glucose deprivation (OGD) followed by reoxygenation; SOD, Superoxide dismutase; TfR2, iron uptake transporter; Tf, transferrin; TfR, transferrin receptor; TRPC6, transient receptor potential canonical 6.)

Compounds	Experimental model	Findings	Mode of action	Refs.
Vitexin	MCAO/Rrat	↓Brain infracted volume;↓the normal histopathology and mitochondrial function;↑activation of Nrf2;↑Keap1/Nrf2/HO‐1	↑Nrf2/SLC7A11/GPX4	[389]
Vitexin	OGD/R neuron cell	↑Cell viability;↓cell apoptosis;↓generation of lipid ROS;↓neuronal cell ferroptosis;↑expressions of Keap1/Nrf2/HO‐1	↑Nrf2/SLC7A11/GPX4	[389]
Calycosin	Transient tMCAO/R/rats	↓Neurological deficits;↓brain edema;↓blood‐brain barrier (BBB) breakdown;↓infarction volume,;↓ neuronal injuries	↓ACSL4;↑GPX4;↓Fe^2+^	[390]
Calycosin	OGD/R/PC12 cells.	↑ Cell viability;↓iron accumulation;↓MDA;↑SOD;↓ROS;↓ACSL4;↓TfR1;↑FTH1;↑GPX4	↓ACSL4;↑GPX4;↓Fe^2+^	[390]
Danhong injection	Permanent MCAO/Rrat	↓Infarct area;↓brains damage;↓ iron accumulation;↑SATB1/SLC7A11/HO‐1	↑SATB1/SLC7A11/HO‐1	[391]
Danhong injection	OGD/HT22 cells	↑Cell viability;↑SATB1/SLC7A11/HO‐1	↑SATB1/SLC7A11/HO‐1	[391]
Ferrostatin‐1	MCAO/Rrat	↓Infarct volume;↑neurobehavioral outcomes;↓iron and MDA;↑GSH;↑SLC7A11;↑GPX4 ↑phosphorylated AKT and GSK3*β*	↑SLC7A11/GPX4;↓Fe^2+^	[392]
Icariside II	MCAO/mice	↓Neurological deficits and sensorimotor function;↓ infarct volume; ↑HO‐1, NQO‐1, SIRT5 and GPX4;↑I*κ*B*α*;↓ phosphorylation level and activity of NF‐*κ*B p65;↓mitochondrial ROS;↓MDA;↓ iron content; ↑NADPH/NADP^+^ ratio, RCI, ATP, GPX4 level, SOD2 activity, and SIRT5 activity;↓the number of GFAP‐positive cells;↓release of IL‐1*β*, IL‐6 and TNF‐*α*	↑GPX4 axis	[393]
Icariside II	OGD/primary astrocytes	↑Cell viability;↓LDH level;↓mitochondrial dysfunction;↓mitochondrial ROS and iron content, and increased the RCI and SIRT5 activity;↓IL‐1*β*, IL‐6, TNF‐*α* and the activity of NF‐*κ*Bp65; ↑ I*κ*B	↑GPX4 axis	[393]
Carthamin yellow	MCAO/rats	↓Neurological deficit score;↓ brain water content;↓ infarct area; ↑ MAP‐2 immunoreactivity in the cortex in MCAO model rats; ↓ MDA;↑SOD;↓Fe^2+^ content;↓TfR1; ↓ACSL4; ↑GPx4;↑FTH1;	↑GPX4 axis	[251]
Galangin	Transient global ischemia/bilateral CCA were occluded	↓Cognitive impairment;↓ neuron death;↓APP in the hippocampus;↓ MDA;↑GPX4;↑SOD activity;↓Fe^2+^ content; ↓Ptgs2 mRNA; ↓4‐HNE;↑GPX4, H2AX and SLC7A11 in the hippocampi	↑SLC7A11/GPX4	[281]
Galangin	Hippocampal neurons/OGD/R	↑Cell viability;↑GPX4 and SLC7A11	↓Fe^2+^	
Rehmannioside A	MCAO/rats	↓Cognitive impairment;↓neurological deficits;↓cerebral infarction;↑p‐PI3K, p‐Akt, Nrf2, HO‐1 and SLC7A11	↑Nrf2/GPX4	[283]
Dimethyl fumarate	Chronic cerebral hypoperfusion/rats	↓Cognitive deficits;↓ hippocampus neuronal damage and loss; ↓ IL‐1*β*, TNF‐*α*, and IL‐6 in hippocampus;↓MDA;↑GSH and SOD; PTGS2;↑FTH1 and xCT; ↓iron content; ↑HO‐1, NQO1 and GPX4;↑Nrf2	↓ ACSL4	[279]
Selenium	Transient MCAO/rats	↑Survival rate of mice;↓infarct area;↓MDA;↑SOD;↑GPX4;↓NOX2	↓LPO;↓Fe^2+^	[284]
Selenium	OGD/R/N2a	↑Cell viability;↓MDA;↓Iron content;↑GSH;↑SOD;↑GSH/GSSG;↑FTH1 mRNA;↓COX2 mRNA; ↑GPX4;↓NOX2	↑GPX4	[284]
Carvacrol	Transient MCAO/rats	↑Memory and learning abilities;↓hippocampus impairment;↓ROS;↓Iron content;↑GPX4 and Fpn1; ↓TFR1	↓ ACSL4;↑GPX4	[249]
Carvacrol	OGD/R/Hippocampal neurons	↑Cell viability;↑GPx4;↓Iron content; ↓MDA	↑GPX4;↓LPO;	[249]
Roots of Astragalus propinquus Schischkin	Transient MCAO/rats	↓Infarct size and neuronal injury;↓Fn, FTH1, FLC, Tf, TfR, DMT1, and TRPC6;↑FPN1 through a Tf/TfR;↑GPX4 and SLC7A11;↑Nrf2	↓Fe^2+^	[252]
Extract of Naotaifang	MCAO/rats	↓Neurobehavioral scores;↑number of Nissl bodies;↓TFR1 and DMT1;↓ROS, MDA and iron; ↑SLC7A11, GPX4 and GSH	↑SLC7A11/GPX4	[250]
*β*‐Caryophyllene	Transient MCAO/rats	↓mNSS neurological scores;↓ infarct volume;↓ pathological features;↑GPX4;↓COX2; ↓ACSL4; ↑NRF2/HO‐1	↑GPX4	[275]
*β*‐Caryophyllene	OGD/R/Primary astrocytes	↑Cell viability;↓ROS generation and iron accumulation;↑GPX4;↓COX2;↑Nrf2/HO‐1	↑Nrf2/GPX4	[275]
Dexmedetomidine	Transient MCAO/mice	↓Neurobehavioral scores;↓infarct volume; ↓MDA;↓Fe^2+^;↓TFR1;↑GSH;↑SLC7A11 and GPX4; ↓mitochondrial damage; ↑Nrf2	↑Nrf2/SLC7A11/GPX4	[253]
Edaravone	Transient MCAO/rats	↓Neurobehavioral scores;↓cerebral infarct volume;↓IL‐6, IL‐1*β*, TNF‐*α*;↓MDA;↓Fe^2+^;↑ GSH;↑Nrf2, GPX4, and FPN	↑Nrf2/GPX4	[280]
Baicalein	Transient MCAO/mice	↓Infarct size and neuronal injury;↓mNSS neurological scores;↓ROS;↓Fe^2+^;↓MDA;↑ GPX4, ACSL3 and xCT; ↓ACSL4; ↑FTH and mitochondrial ferritin (FTMT)	↑GPX4;↓LPO;↓Fe^2+^	[276]
Baicalein	RSL3/HT22 cells	↑Cell viability;↓Fe^2+^; smaller volume, higher mitochondrial membrane electron density and disrupted mitochondrial cristae were ameliorated by baicalein;↓ROS;↑MMP, GSH;↓MDA	↑GPX4;↓LPO;↓Fe^2+^	[276]
Baicalein	OGD/R/HT22 cells	↑Cell viability;↓ROS;↓cell death		[276]
HYSA and HYSB	OGD/R/PC12 cells	↑Cell viability;↓Fe^2+^;↓MDA;↓4‐HNE;↑GSH/GSSG, SLC7A11, GPX4	↑SLC7A11/GPX4;↓LPO;↓Fe^2+^	[282]
Compound Tongluo Decoction	Transient MCAO/rats	↓Infarct size and neuronal injury;↓ER stress‐related proteins GRP78 and XBP‐1s;↓ATF4, PERK and cleaved. caspase 3; ↓MDA;↓ROS;↑SOD;↓ACSL4;↓ALOX5;↑GPX4;↑angiogenesis; ↑Sonic Hedgehog pathway	Sonic Hedgehog pathway	[266]
Compound Tongluo Decoction	OGD/R/PC12 cells	↑Cell viability;↓ACSL4;↓ALOX5;↑GPX4;↓ER stress‐related proteins GRP78 and XBP‐1s;↓ATF4, PERK and cleaved caspase 3; ↑Sonic Hedgehog pathway	Sonic Hedgehog pathway	[266]
Resveratrol	Transient MCAO/rats	↓Infarct size and neuronal injury;↓ACSL4;↑GPX4;↑ferritin	↓ACSL4;↑GPX4	[277]
Resveratrol	OGD/R/primary cortical neurons	↑Cell viability;↓ACSL4;↑GPX4;↓Fe^2+^;↑GSH;↑ferritin;↑maintain mitochondrial structure	↓ACSL4;↑GPX4;↓Fe^2+^	[277]

**Table 5:** Emerging compounds targeting key regulators of ferroptosis to attenuate ICH (ACSL4, long‐chain acyl‐CoA synthetase 4; ALOX5, arachidonate 5‐lipoxygenase; DMT1, iron importer divalent metal transporter 1; FPN1, iron efflux transporter; FAC, ferric ammonium citrate; FSP1, ferroptosis suppressor protein 1; LPO, LPO; OHSCs, organotypic hippocampal slice cultures; PIH, Pyridoxal isonicotinoyl hydrazine; PCN, primary cortical neurons.)

Compounds	Study models	Effects	Target	Refs.
Dexmedetomidine	Collagenase‐induced ICH/mice	↓Neurological deficits;↓brain water content;↓hemorrhagic lesion volume;↓cytoplasmic microvacuolation and nuclear pyknosis;↓TfR1;↑GPX4;↓mitochondria swelled;↓membrane ridges disappeared;↓cytoplasmic vacuolization	↑GPX4	[326]
Dexpramipexole	Hemoglobin/mice	↑Locomotion and Motor Coordination Recovery;↓Hematoma Volume;↓white matter damage;↓ROS;↓Fe^2+^;↓ MDA;↑GPX4;↑FSP1	↓Fe^2+^;↑GPX4	[310]
Withaferin A	Caudate‐putamen (CPu) injection of autologous blood in mice	↓Brain tissue injury;↓iron deposition;↑neurological function;↓MDA;↑SOD and GPX4;↑Nrf2/HO‐1	Nrf2/GPX4	[394]
Withaferin A	SH‐SY5Y cells/hemin	↓Cell injury;↓MDA;↑SOD and GPX4;↑Nrf2/HO‐1; ferrostatin‐1 reduced hemin‐induced SH‐SY5Y neuronal cell injury	Nrf2/GPX4	[394]
Pioglitazone	Striatum injection of autologous blood/rat	↑Clearance of hematoma;↓ brain edema;↑ recovery nerve function;↑PPAR*γ*, Nrf2 and GPX4	Nrf2/GPX4	[316]
Pioglitazone	Rat primary hippocampal neurons/hemin	↑Survival rate of neurons;↓ MDA;↓ 4‐HNE;↑GSH;↑PPAR*γ*, Nrf2 and GPX4	Nrf2/GPX4	[316]
Crocin	Caudate‐putamen (CPu) injection of autologous blood/mice	↓Brain edema and neurological deficits;↑SOD and GPX;↓ MDA;↓Fe2+;↑GPX4;↑FTH1;↑ SLC7A11;↑Nrf2	Nrf2/SLC7A11/GPX4	[311]
Vildagliptin	Collagenase type VII micro‐injection into the right basal ganglia/mice	↑Neurological deficit scores;↓hematoma volume;↓degeneration of neurons; ↓activation of microglia/macrophages;↓infiltration of neutrophil;↓Fe^2+^;↑GPX4;↓ MDA	GPX4	[312]
Dauricine	Collagenase‐induced ICH/mice	↓ Neurological deficits;↓brain water content;↓Fe^2+^;↑GPX4;↑GSR	↓Fe^2+^;↓LPO	[309]
(‐)‐Epicatechin	Collagenase‐induced ICH/mice	↓Brain injury volume;↑neurologic function;↓MDA;↓oxidative injury;↑SOD;↓HO‐1 in the hemorrhagic hemisphere;↓Fe^2+^; lipocalin‐2 (LCN2)	↓Fe^2+^;↓LPO	[306]
Paeonol	Hemin/HT22	↓MDA, ROS;↑GSH;↓Fe^2+^;↓ACSL4	↓Fe^2+^;↓ACSL4;↓LPO	[308]
Paeonol	Hemin/PCN	↓MDA, ROS;↑GSH;↓Fe^2+^;↓ACSL4	↓Fe^2+^;↓LPO;↓ACSL4	[308]
Paeonol	Collagenase‐induced ICH/mice	↓Neurological severity score;↓MDA;↓Fe^2+^;↓ACSL4	↓Fe^2+^;↓LPO;↓ACSL4	[308]
Isorhynchophylline	Mouse hippocampal HT‐22/FAC	↑Cell viability; ↑GPX4;↑SOD; ↑FPN1;↓ HNE;↓ MDA; ↓Fe^2+^; ↑SLC7A11	↓Fe^2+^;↓LPO	[318]
HET0016; 20–6,15‐HEDGE	Hemoglobin‐treated OHSCs	↑GPX;↓cell death, iron deposition, and lipid ROS	↓LPO	[307]
Curcumin Nanoparticles	Collagenase‐induced ICH/mice	↓Hematoma volume;↑GPX4;↑NRF2/HO‐1	↑GPX4;↓NRF2/HO‐1	[395]
HET0016	Collagenase‐induced ICH/mice	↓Focal deficits;↓lesion volume;↓ iron accumulation; ↑GPX4;↑GSH;↓ MDA and HNE	↑GPX4;↓LPO	[307]
PIH	Collagenase‐induced ICH/mice	↓Neurological deficit scores;↓ROS production;↓iron accumulation;↓LPO;↑GPX4;↓ COX2;↓ IL‐1*β*;↓ TNF‐*α*	↑GPX4;↓LPO	[317]
Selenium	Hemin/PCN	↑Cell viability;↑GPX4;	↑GPX4	[263]
Selenium	Collagenase‐induced ICH/mice	↑Functional Recovery;↓Hematoma size;↑GPX4	↑GPX4	[263]
Baicalin	Hemin/PC12	↑Cell viability;↑GPX4;↑SLC7A11	↑GPX4	[325]
Baicalin	RSL3/PC12	↑Cell viability;↑GPX4;↓ROS;	↑GPX4	[325]
Baicalin	PC12/erastin	↑Cell viability;↑SLC7A11;↓ROS;	↑GPX4	[325]
Baicalin	PCN/hemin‐ or erastin	↑Cell viability;↑SLC7A11;↓ROS;	↑GPX4	[325]
Baicalin	Collagenase‐induced ICH/mice	↓Motor deficits;↓hemorrhagic lesion;↑GPX4;↑SLC7A11;↑SLC3A2; ↑TFR; ↓DMT1	↑GPX4	[325]
PIH	Erastin‐treated PC‐12 cells	↓Neuronal cell death;↓ LPO	↓LPO	[317]
Isorhynchophylline	Collagenase‐induced ICH/SD rat	↑mNSS score;↓brain water content;↓Blood‐Brain Barrier permeability; ↑GPX4;↑SLC7A11;↓ HNE;↓ MDA;↓ lipid ROS;↓ p53	↓LPO	[318]
N‐Acetylcysteine	Collagenase‐induced ICH/ALOX5 KO mice	↑Functional recovery;↓hematoma size;↓brain edema;↓ALOX5	↓LPO	[396]
N‐Acetylcysteine	PCN/hemin	↓Neuronal death	↓LPO	[396]
Resveratrol	HT‐22/erastin	↑Cell viability;↓ROS;	↓LPO	[397]
Resveratrol	Collagenase‐induced ICH/SD rat	↓Motor deficits;↓hemorrhagic lesion	↓LPO	[397]

### 5.1 Inhibition of Ferroptosis to Alleviate PD

Quercetin,^[^ ^350^ ^]^ Dl‐3‐n‐butylphthalide,^[^ ^109^, ^351^ ^]^ *β*‐hydroxybutyric acid,^[^ ^352^ ^]^ thonningianin A,^[^ ^112^ ^]^ GW501516 (a specific PPAR*δ* agonist),^[^ ^111^ ^]^ hinokitiol,^[^ ^113^ ^]^ paeoniflorin,^[^ ^125^ ^]^ *α*‐Lipoicacid,^[^ ^114^ ^]^ iron chelator deferoxamine (DFO),^[^ ^353^ ^]^ SK4/DFO,^[^ ^354^ ^]^ clioquinol,^[^ ^115^ ^]^ apoferritin,^[^ ^126^ ^]^ ferrostatin‐1,^[^ ^110^, ^355^ ^]^ SK4/DFO,^[^ ^354^ ^]^ and idebenone^[^ ^356^ ^]^ alleviate PD through inhibiting ferroptosis (Table 1).

### 5.2 Inhibition of Ferroptosis to Alleviate AD

Salidroside,^[^ ^357^, ^358^ ^]^ edaravone,^[^ ^359^ ^]^ senegenin,^[^ ^360^ ^]^ tetrahydroxy stilbene glycoside (TSG),^[^ ^361^ ^]^ ginkgolide B,^[^ ^362^ ^]^ forsythoside A,^[^ ^363^ ^]^ deferoxamine,^[^ ^364^ ^]^ insamgobonhwan,^[^ ^365^ ^]^ *γ*‐glutamylcysteine (*γ*‐GC),^[^ ^366^ ^]^ a novel lipoic acid‐niacin dimer N2L,^[^ ^367^ ^]^ eriodictyol,^[^ ^368^ ^]^ selenium (Se),^[^ ^369^, ^370^ ^]^ clioquinol,^[^ ^371^ ^]^ GW7647,^[^ ^372^ ^]^ *α*‐Lipoic acid,^[^ ^373^ ^]^ OABL,^[^ ^374^ ^]^ CMS121,^[^ ^375^ ^]^ Fer‐1,^[^ ^173^ ^]^ Lip‐1,^[^ ^173^ ^]^ coenzyme Q10,^[^ ^376^ ^]^ 7,8‐dihydroxyflavone (7,8‐DHF),^[^ ^377^ ^]^ 2′,6′‐dihydroxy‐4′‐methoxy dihydrochalcone (DHMDC),^[^ ^378^ ^]^ kojic acid,^[^ ^379^ ^]^ crocin,^[^ ^380^ ^]^ celecoxib,^[^ ^381^ ^]^ allicin,^[^ ^382^ ^]^ centella asiatica,^[^ ^383^ ^]^ and ellagic acid^[^ ^384^ ^]^ alleviate AD through inhibiting ferroptosis (Table 2).

### 5.3 Inhibition of Ferroptosis to Alleviate Brain Injury after ALS

Fer‐1,^[^ ^239^, ^243^ ^]^ liproxstatin‐1,^[^ ^239^ ^]^ vitamin E,^[^ ^238^, ^385^ ^]^ deferoxamine,^[^ ^385^ ^]^ salicylaldehyde isonicotinoyl hydrazone,^[^ ^224^ ^]^2‐(2‐Hydroxyphenyl)‐benzoxazole,^[^ ^386^ ^]^ VAR10303^[^ ^387^ ^]^ deferiprone^[^ ^243^, ^388^ ^]^ alleviate brain injury after ALS through inhibiting ferroptosis (Table 3).

### 5.4 Inhibition of Ferroptosis to Alleviate Brain Injury after IS

Vitexin,^[^ ^389^ ^]^ calycosin,^[^ ^390^ ^]^ danhong injection,^[^ ^391^ ^]^ Fer‐1,^[^ ^392^ ^]^ icariside II,^[^ ^393^ ^]^ carthamin yellow,^[^ ^251^ ^]^ galangin,^[^ ^281^ ^]^ rehmannioside A,^[^ ^283^ ^]^ dimethyl fumarate,^[^ ^279^ ^]^ selenium,^[^ ^284^ ^]^ carvacrol,^[^ ^249^ ^]^ roots of astragalus propinquus schischkin,^[^ ^252^ ^]^ extract of Naotaifang,^[^ ^250^ ^]^ *β*‐caryophyllene,^[^ ^275^ ^]^ dexmedetomidine,^[^ ^253^ ^]^ edaravone,^[^ ^280^ ^]^ baicalein,^[^ ^276^ ^]^ hydroxysafflor yellow A(HYSA) and hydroxysafflor yellow B(HYSB),^[^ ^282^ ^]^ compound tongluo decoction,^[^ ^266^ ^]^ and resveratrol^[^ ^277^ ^]^ alleviate brain injury after IS through inhibiting ferroptosis (Table 4).

### 5.5 Inhibition of Ferroptosis to Alleviate Secondary Brain Injury after ICH

Dexmedetomidine,^[^ ^326^ ^]^ dexpramipexole,^[^ ^310^ ^]^ withaferin A,^[^ ^394^ ^]^ pioglitazone,^[^ ^316^ ^]^ crocin,^[^ ^311^ ^]^ vildagliptin,^[^ ^312^ ^]^ dauricine,^[^ ^309^ ^]^ (‐)‐Epicatechin,^[^ ^306^ ^]^ paeonol,^[^ ^308^ ^]^ isorhynchophylline,^[^ ^318^ ^]^ arachidonic acid metabolite 20‐hydroxyeicosatetraenoic acid (20‐HETE) synthesis inhibitor N‐hydroxy‐N′‐(4‐n‐butyl‐2‐methylphenyl)‐formamidine (HET0016) and 20‐6,15‐HEDGE (20‐HETE antagonist),^[^ ^307^ ^]^ curcumin nanoparticles,^[^ ^395^ ^]^ pyridoxal isonicotinoyl hydrazine (PIH),^[^ ^317^ ^]^ selenium,^[^ ^263^ ^]^ baicalin,^[^ ^325^ ^]^ isorhynchophylline,^[^ ^318^ ^]^ N‐acetylcysteine,^[^ ^396^ ^]^ and resveratrol^[^ ^397^ ^]^ alleviate secondary brain injury after ICH through inhibiting ferroptosis (Table 5).

### 5.6 Inhibition of Ferroptosis to Alleviate Early Brain injury After SAH

Astragaloside IV,^[^ ^335^ ^]^ PKR inhibitor C16,^[^ ^347^ ^]^ netrin‐1,^[^ ^398^ ^]^ quercetin,^[^ ^399^ ^]^ taurine,^[^ ^400^ ^]^ puerarin,^[^ ^345^ ^]^ an inhibitor of inducible nitrite oxide synthase L‐NIL,^[^ ^401^ ^]^ resveratrol and selisistat,^[^ ^348^ ^]^ cepharanthine,^[^ ^344^ ^]^ baicalin,^[^ ^334^ ^]^ Fer‐1,^[^ ^333^, ^402^ ^]^ and Lip‐1^[^ ^346^ ^]^ alleviate early brain injury after SAH through inhibiting ferroptosis (**Table** **6**).

**Table 6:** Emerging compounds targeting ferroptosis to attenuate EBI‐SAH (ACSL4, long‐chain acyl‐CoA synthetase 4; DMT1, iron importer divalent metal transporter 1; L‐NIL, an inhibitor of inducible nitrite oxide synthase)

Compounds	Experimental model	Findings	Mode of action	Ref
Astragaloside IV	Endovascular filament perforation model/rat	↓Early brain damage after SAH;↓Fe^2+^, MDA, and Lipid ROS; ↑GSH; ↑GPX4 and SLC7A11;↑Nrf2/HO‐1	↑Nrf2/SLC7A11/GPX4	[335]
PKR inhibitor C16	Internal carotid artery puncture/rat	↓Severe fundic hemorrhage;↓neurological impairment;↓MDA;↓iron ion accumulation;↑GPX4 and FTH1 levels in rats	↑GPX4;↓ LPO	[347]
Netrin‐1	Endovascular filament perforation/mice	↑Cell viability;↑GPX4; ↓Lipid ROS; ↑Nrf2; ↑GPX4; ↑CoQ10‐FSP1	↑Nrf2/GPX4; ↑CoQ10‐FSP1	[398]
Netrin‐1	Hemin/HT‐22 cells	↑Survival probability, greater survival of neurons, and neurological score; ↑PPAR*γ*; ↑Nrf2; ↑GPX4; ↑CoQ10‐FSP1	↑Nrf2/GPX4; ↑CoQ10‐FSP1	[398]
Quercetin	Internal carotid artery puncture/rat	↑Neurological function;↑GPX4, xCT, and FPN1;↓TfR1;↓iron accumulation;↓lipid peroxidation in the cortex of SAH rats	↑xCT/GPX4;↓ LPO	[399]
Taurine	Internal carotid artery puncture/rat	↓Neurological impairment;↓oxidative stress;↓iron accumulation;↑BBB integrity;↓neuronal ferroptosis	↑xCT/GPX4	[400]
Taurine	Hemin/HT‐22 cells	↓MDA levels;↓ ROS accumulation;↑ SLC7A11 and GPX4;↑AKT/GSK3*β*	↑xCT/GPX4	[400]
Puerarin	Endovascular filament perforation model/rat	↓Short‐Term Neurobehavioral Deficits;↑pAMPK, PGC1*α*, Nrf2, HO‐1, SOD, GPX4, and GSH;↓4‐HNE, MDA, ACSL4, GSSG and iron concentration in the ipsilateral hemisphere at 24 h after SAH.	AMPK/PGC1*α*/Nrf2/GPX4	[345]
L‐NIL	Prechiasmatic cistern injection rat model	↓Number of M1 microglia;↓neuroinflammation;↓neurobehavioral deficits;↓brain edema and neuronal injury;↑outcomes of neurological function;↑ferroptosis of M1 microglia	‐	[401]
Resveratrol and selisistat	Prechiasmatic cistern injection mouse model	↓MDA;↑GPX4 and FSP1	↑GPX4;↓ LPO	[348]
Resveratrol and selisistat	Oxyhemoglobin (oxyHb)/HT‐22 cells	↓MDA;↑GPX4 and FSP1	↑GPX4;↓ LPO	[348]
Cepharanthine	Endovascular perforation/mice	↓SHA grade;↑neurological performance evaluated by the modified Garcia scale;↓brain edema, and BBB disruption;↓LPO (MDA and 4‐HNE);↑GSH;↓ALOX15 in Endothelial Cells and Microglia	↓ LPO	[344]
Cepharanthine	RSL3 and hemin/bEend.3 endothelial cells and BV2 microglial cells	↑Cell viability of bEnd.3 cell line;↓RSL3‐induced lipid ROS accumulated in endothelial cells	↓LPO	[344]
Baicalin	Autologous femoral arterial blood was injected into a prechiasmatic cistern/rat	↓Brain edema;↑beam balance scores and modified Garcia scores;↓Fe^2+^, malondialdehyde, and ROS levels in the brain;↑GSH;↓beclin1, LC3‐II, and LC3‐I protein levels	↑GPX4; ↓LPO	[334]
Ferrostatin‐1	Endovascular filament perforation/rat	↓Blood‐brain barrier impairment, brain edema, behavioral deficits, and neuronal damage; ↑SLC7A11 and GPX4;↓damage‐associated molecular pattern molecules and inflammatory cytokines; p53 inhibitor pifithrin‐*α* could significantly block cortical SAH‐induced ferroptosis	↑SLC7A11/GPX4	[402]
Liproxstatin‐1	Endovascular filament perforation/rat	↓Neurological deficits and brain edema, neuronal cell death, and restored the redox equilibrium; after SAH;↑GPX4;↓ACSL4 and COX2;↓activation of microglia and the release of IL‐6, IL‐1*β*, and TNF‐*α*	↓Iron;↑GPX4;↓LPO	[346]
Fer‐1	Oxyhemoglobin/SH‐SY5Y	↑Cell viability;↓iron;↓TfR1;↓LPO	↓Iron;↓LPO	[333]
Fer‐1	Endovascular perforation/rats	↓Neurological scores;↓blood‐brain barrier permeability;↓brainedema;↑Fpn;↓iron;↓LPO;↑GPX4;↑GSH	↓Iron;↑GPX4;↓LPO	[333]

## 6 Conclusions and Perspectives

In conclusion, this review article summarizes the recent progress of understanding the pathological pathways and regulatory mechanisms of ferroptosis in neurological diseases including PD, AD, ALS, IS, ICH, and SAH. We discuss the application of ferroptosis inhibitors in mitigating neurodegenerative diseases and provide a new target for future treatment and prevention of these diseases through targeting ferroptosis. Ferroptosis is involved in the neurodegeneration of neurological diseases including PD, AD, ALS, IS, ICH, and SAH. Ferroptosis is involved in the pathogenesis of neurodegeneration, while stroke leads to ferroptosis, resulting in secondary brain injury. This review highlights the promising potential of pharmacological inhibition of ferroptosis for the treatment of neurological diseases. However, the current research on the role of ferroptosis in neurological diseases is still poorly understood. Despite many advances, relatively little is known about how ferroptosis orchestrates diverse cellular events. Firstly, the regulatory mechanism underlying ferroptosis in neurological diseases needs to be uncovered. Secondly, we should note that ferroptosis plays important roles in multiple diseases besides neurological diseases, and inhibition of ferroptosis in neurological diseases as a therapeutic might result in drug resistance in cancer. Therefore targeting ferroptosis maybe a double‐edged sword, and lead to unexpected toxicity and injury. Third, the specific biomarker for ferroptosis is urgent needed for accurate predicting the efficiency of inhibition of ferroptosis. Furthermore, most data obtained from experimental studies are far from reaching impact on clinical application and more clinical approaches are necessary. Nevertheless, targeting ferroptosis will bring new directions for the treatment of neurological diseases.

## Conflict of Interest

The authors declare no conflict of interest.

## Author Contributions

Y.W. and S.W. contributed equally to this work. Y.W., S.W., and Q.L. conducted the analytical part. Y.W., S.W., and H.W. wrote the first version of the manuscript. Y.W., S.W., and H.W. downloaded the reference and processed the graph and the table in the manuscript. H.S. and H.W. conceived and coordinated the study, and critically evaluated the data. All authors read and approved the final manuscript.
